# Mechanics to pre-process information for the fine tuning of mechanoreceptors

**DOI:** 10.1007/s00359-019-01355-z

**Published:** 2019-07-03

**Authors:** Friedrich G. Barth

**Affiliations:** Department of Neurobiology, Faculty of Life Sciences, University of Vienna, Althanstr.14, 1090 Vienna, Austria

**Keywords:** Mechanoreception, Pre-processing of information, Auxiliary structures, Stimulus transformation, Sensor fine tuning

## Abstract

Non-nervous auxiliary structures play a significant role in sensory biology. They filter the stimulus and transform it in a way that fits the animal’s needs, thereby contributing to the avoidance of the central nervous system’s overload with meaningless stimuli and a corresponding processing task. The present review deals with mechanoreceptors mainly of invertebrates and some remarkable recent findings stressing the role of mechanics as an important source of sensor adaptedness, outstanding performance, and diversity. Instead of organizing the review along the types of stimulus energy (force) taken up by the sensors, processes associated with a few basic and seemingly simple mechanical principles like lever systems, viscoelasticity, resonance, traveling waves, and impedance matching are taken as the guideline. As will be seen, nature makes surprisingly competent use of such “simple mechanics”.

## Introduction

Nervous systems process data initially taken up as meaningless and abstract stimuli. They turn them into signals, cues, and complex information, thereby giving them biological meaning in a behavioral context. Whatever the refinement of data processing in the central nervous system (CNS) may be, it will to a large extent always be at the mercy of the sensory organs found at the interface between the organism and its habitat. Recent research underlines the remarkable “cleverness” often found in the sensory periphery and its relevance in preventing the CNS from being overwhelmed by the amount of sensory input and a corresponding processing task. Much of this “cleverness” comes from the non-nervous auxiliary structures. Their eminent role is not restricted to the arthropods, but may be particularly relevant for them, considering their small brains and a comparatively limited capacity of processing and integrating data.

The present review concentrates on mechanosensors. These take up and respond to stimuli representing different kinds of force, like touch, medium flow, airborne sound, substrate vibrations, and strain. The idea is to consider behaviorally important parameters contained in the mechanical stimulus and to ask, which mechanical “trick” is applied to deal with it properly, streamlining the sensor’s performance in regard to the receiver’s needs. The dominant concept underlying the relevance of this question is an adaptedness of the sensors and their filtering properties, which assures the necessary selectivity and the adequate handling of the biologically relevant stimulus patterns. This type of approach chosen for the present review may also bio-inspire the engineer interested in designing sensors based on principles successfully at work in nature. Emphasis will be on the mechanical pre-processing of a few mechanoreceptors with highly refined filter properties evolved to match particular biological needs. With its focus on mechanical attributes of non-nervous stimulus conducting structures, the present essay will discuss lever systems, viscoelasticity, resonance, impedance matching, and other aspects, which often strongly determine sensor absolute sensitivity, frequency selectivity, the time course of the receptor response, directional sensitivity, the signal-to-noise ratio, and other physiologically relevant sensor properties.

Obviously, not all aspects of sensory mechanics can be covered here. The review is not meant to be encyclopedic and also cannot do full justice to the ensemble of all complex mechanical interactions at work in a particular sensor. Instead, the relevance of mechanical pre-processing shall be emphasized by presenting selected aspects of exemplary mechanoreceptors with an emphasis on arthropods. A few well-studied classical vertebrate cases will shortly be reviewed to highlight the relevant problem and background. It will become clear that although some of the physics addressed may appear trivial at first sight, its biological implementation often turns out to be not trivial at all.

## The power of lever systems

### Hair-like arthropod sensors, rodent whiskers, and the vertebrate middle ear

As we have learned at school about joints and bones, our skeletal system and that of other vertebrates represent a system of levers. Depending on the arrangement of the axis of rotation, the location of the muscle attachment sites, and the length ratio of the power-and-load arms in such a system, it is predominantly used for transmitting/generating either force or displacement. This allows the adjustment of the simplest “mechanical machine” to many different tasks.

#### Arthropod “hairs”

**Fig. 1:**
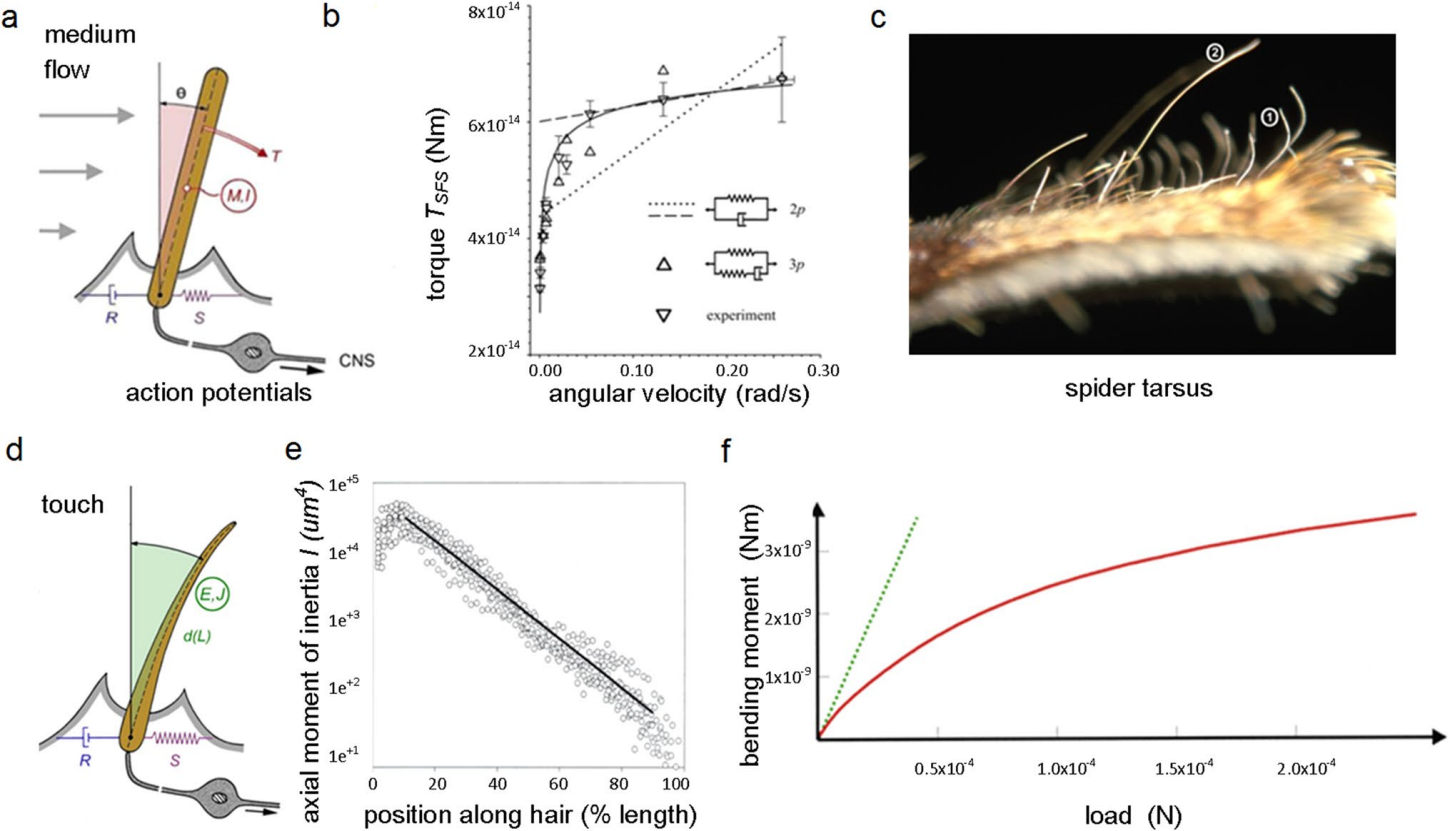
Lever systems: **a** Schematic of an arthropod medium-flow-sensitive hair sensillum; *T* torque, *M* mass, *I* inertia, *R* viscosity, *S* elasticity, *CNS* central nervous system, and θ deflection angle. Different lengths of arrows indicate difference in medium-flow velocity due to boundary layer. **b** Torque resisting the deflection of a spider (*Cupiennius salei, C.s.*) trichobothrium when deflected at different speed; inset shows two- and three-parameter model representing the mechanical properties of the hair’s suspension. **c** Tarsus (length c. 5 mm) of a spider leg (*C.s.*) showing air flow sensitive trichobothrium (1) and tactile hair (2). **d** Schematic of arthropod tactile hair-like sensillum; *E* Young´s modulus, *J* second moment of inertia, *L* length of hair, *R* viscosity, *S* elasticity. **e** Axial moment of inertia along the tactile hair of a spider (*C.s*.). **f** Bending moment (red) of spider (C.s.) tactile hair exposed to increasing stimulus force from above; green line indicates values calculated for stiff hair. (**a** Barth 2012a, b, c; **b** McConney et al. 2009; **c**, **d** Barth 2002; **e**, **f** Dechant et al.^2001^); with kind permission from Elsevier Ltd. (**a**, **c**, **d**), the Royal Society London (**b**), and Springer-Verlag GmbH (**e**, **f**)

Like mammals, for which true hairs are a defining feature, many arthropods are covered by cuticular hair-like structures. Typically, these “hairs” show high-aspect ratios; the majority of them is innervated and mechanosensitive to the deflection of the “hair” shaft. Their axis of rotation, the pivot, is located at an articulation of varying stiffness close to their base and only micrometers below the surface of the cuticular exoskeleton. All these “hairs” represent first-order lever arms, with the pivot lying between the power (effort) and the load (resistance) and between the long and the short lever arms, respectively (Fig. 1).

##### Lever arms

Typically, in arthropod medium-flow mechanoreceptors, the outer lever arm, which is exposed to the stimulus, is much longer than the inner one, which is coupled to the sensory cell (Fig. 1a, c, d). The corresponding length ratios reach values of several hundreds or even more than 1000 (Keil 1997a, b; Shimozawa et al. 1998; Barth 2002, 2014, 2016). Therefore, displacement at the tip of the hair is strongly scaled down at the inner lever arm and the site of the sensory cell dendrite attachment. According to experimental evidence, the displacement at the lowest threshold measured (0.01° deflection) needed for a physiological response of the sensory cell is as small as 0.07 nm in spider trichobothria and 7 nm at a deflection of 1° (Barth and Höller 1999). For cricket cercal hairs, a value of c. 30 nm was found for a deflection by 1° (Gnatzy and Tautz 1980). Thurm (1982) calculated a change of dendrite diameter of 0.05 nm only at a threshold movement of the inner lever arm by 0.1 nm. A similarly small value of inner lever arm tip displacement is given by Shimozawa and Kanou (1984). For spider tactile hairs (Fig. 1c, d), the corresponding value was found to be 0.05 µm at a threshold deflection angle of 1° (Albert et al. 2001). Importantly, force is scaled up at the same ratio, the mechanical advantage being given by the ratio of the lengths of the outer and inner lever arms. This underlines the relevance of combining very small displacement and increased force for the transduction process proper, defined by the conductance changes of mechanosensitive membrane channels. Presumably, such small displacements prevent damage at the cell’s transduction site, which would be substantial with strong stimulation (displacement of proximal “dendritic” tip *dp*/displacement at distal hair tip dd = length of proximal lever arm L/length of distal lever arm l; accordingly, force fd/fp = dp/dd).

Bending of the power arm, that is of the hair’s outer lever arm when deflected by external forces (see below) and its relevance for sensing is not only linked to the elastic restoring force at its articulation, but also to the second moment of inertia of the lever arm itself. So far, tapered spider tactile “hairs*”* (diameter decreasing along their length) are the only arthropod case where this was studied in some detail. The most interesting outcome of this study (Dechant et al. 2001; Barth 2016) was the structural and mechanical heterogeneity of the hair shaft. Its second moment of area, *J*, changed by almost four powers of ten along its length from the base to its tip (Fig. 1e). *J* is also referred to as flexural inertia, based on the hair’s cross-sectional geometry and used to calculate its deflection and stress due to the moment or force applied to it. According to both experiment and theory (finite-element analysis), this highly refined distribution of the inertial moment contributes fundamentally to the way which the hair bends under stimulation. It is key to understand the hair’s particular sensitivity to the onset of a stimulus and its protection from mechanical over-stimulation and damage, axial surface stresses never exceeding c. 3 × 10^5^ Nm^−2^ (Fig. 1f) (Dechant et al. 2001).

##### Lever arm and suspension

In addition to the deflection by external forces, the degree of elasticity (*S*) of the suspension structures and indeed their viscosity (*R*) determine the resistance counteracting hair shaft deflection and, therefore (also depending on the mechanical properties of the hair shaft per se), the amount of bending. Thus, the mechanical properties of the pivot are crucially affecting the lever’s behavior under load. Their physiological relevance is most evident when comparing the two extreme cases of a range of cuticular hairs as found on a wandering spider (*Cupiennius salei*): (1) tactile hairs (review: Barth 2016) responding to direct exposure to relatively strong forces and (2) trichobothria (review: Humphrey and Barth 2008; Barth 2016) responding to the slightest whiff of air, driven by the frictional forces exerted by moving air.

*S* values of the suspension structures differ by up to four powers of ten, with the medium-flow sensor showing values of about 10^−12^ Nm rad^−1^ and the tactile hair showing values of about 10^−9^ to 10^−8^ Nm rad^−1^ (Barth and Dechant 2003). Correspondingly, the frictional forces needed to deflect a trichobothrium at its physiological threshold (eliciting an action potential response) are on the order of 0.4–4 x 10^−6^ N only. The force needed to deflect a tactile hair is c. 5x10^−5^ N/° at small deflections for which they are particularly sensitive. It follows that in terms of absolute sensitivity the two types of hair-like sensors differ greatly. Whereas trichobothria have evolved to be extremely sensitive to air flow (important, e.g., in the context of prey capture; Klopsch et al. 2012, 2013; Barth 2014; insect filiform hairs: Casas et al. 2008); the spider tactile hairs have evolved their mechanical properties to be both sensitive enough to indicate the onset of a tactile stimulus (like most sensors they are more “interested” in changing than in static stimulus conditions) and at the same time to protect the hair from breaking, when stimulated strongly and many thousands of times during a lifetime. The tactile “hair” is, indeed, an “event recorder”, instead of informing the nervous system about details of continuing stimulation. Like the protection from breaking, this property is effectively supported by mechanical “pre-processing” (Fig. 1f) (Albert et al. 2001; Dechant et al. 2001). The stiffness of the pivot substantially resists the hair shaft’s deflection, thereby promoting its bending under stimulation. With the tactile stimulus coming from above the effective lever arm is longest at the onset of the stimulus. With increasing stimulus magnitude, the point of load introduction moves towards the pivot. Thereby the effective lever arm increasingly shortens and the relative torque and thus mechanical sensitivity are reduced. However, at the same time, the mechanical working range is extended and the danger of breaking reduced. There is no evidence that trichobothria bend when deflected by the surrounding air flow. Their hair shaft has a bending stiffness of ca. 0.18 N m^−1^ (McConney et al. 2009).

Interestingly, joint restoring torques differ between individual tactile hairs, whereas the maximum axial stresses and bending are the same. This indicates a fine match between the stiffness of the outer lever arm and that of its suspension (Dechant 2001; Dechant et al. 2001).

#### Rodent whiskers

Similar to the arthropod hair-like mechanosensors, the whisker system of rodents and other mammals works with stimulus transforming vibrissae with mechanosensory cells in their follicle–sinus complex (Ebara et al. 2002; Hartmann et al. 2003; Towal et al. 2012). There is a lot of bending in rat tapered whiskers (apart from their intrinsic curvature) when used for active sensing. Importantly, the morphological variables including global and base curvature and the whisker’s angle reduction after contact (angle absorption) and also whisker angular velocity potentially provide a basis of morphological “coding”, since they are invariant representations of object azimuthal and radial location. The precise distribution of forces in the follicle and thus also the readout by the mechanoreceptors in the follicle are not clear yet, although they must be considered the crucial step in the actual use of the morphological data by the nervous system. In any case, the forces generated by a collision of the whisker and mechanically transmitted to the whisker base will be transformed into small deformations and finally transduced to electrical nervous signals. The readout of the potentially available tactile information seems to be complex. It would have to be scaled by the whisker’s individual shape, its motor variables, the mechanical conditions provided by the follicular blood sinus, and also the position, orientation, and velocity of the animal’s head (Simony et al. 2010; Towal et al. 2012; Bagdasanian et al. 2013).

The effect of varying the length of hair-like sensors on sensitivity and tuning will be discussed further below. It plays an important role both in spider trichobothria and in rat whiskers.

#### Vertebrate middle ear

Regarding the sensory function of lever systems, the ossicular chain in the vertebrate middle ear, in particular that of humans, has been a textbook example for a long time and should at least shortly be mentioned here. It adds to overcome the mismatch between the respective impedances of air and the fluid-filled cochlea (see below) and helps to transform airborne sound to sound pressure and volume velocities in the inner ear **(**Rosowski 2003). The middle ear chain of bones causes a lever action (human ear:malleus lever arm longer than incus lever arm by a factor of 2.1) and multiplies the force acting on the tympanum 1.3 times. The displacement of the tympanum is scaled down, while the force introduced into the inner ear is scaled up by the same ratio. Middle ear morphology greatly differs among the vertebrates as detailed by Rosowski (2003). As we will see further below, apart from the leverage, the ratio of the area taking up the stimulus (ear drum) and that passing it on to the inner ear (stapedial footplate; oval window) are the other crucial components of impedance matching.

## Viscoelasticity and glass transition

### Determining temporal response characteristics, frequency tuning, and absolute sensitivity

#### Response decline

**Fig. 2:**
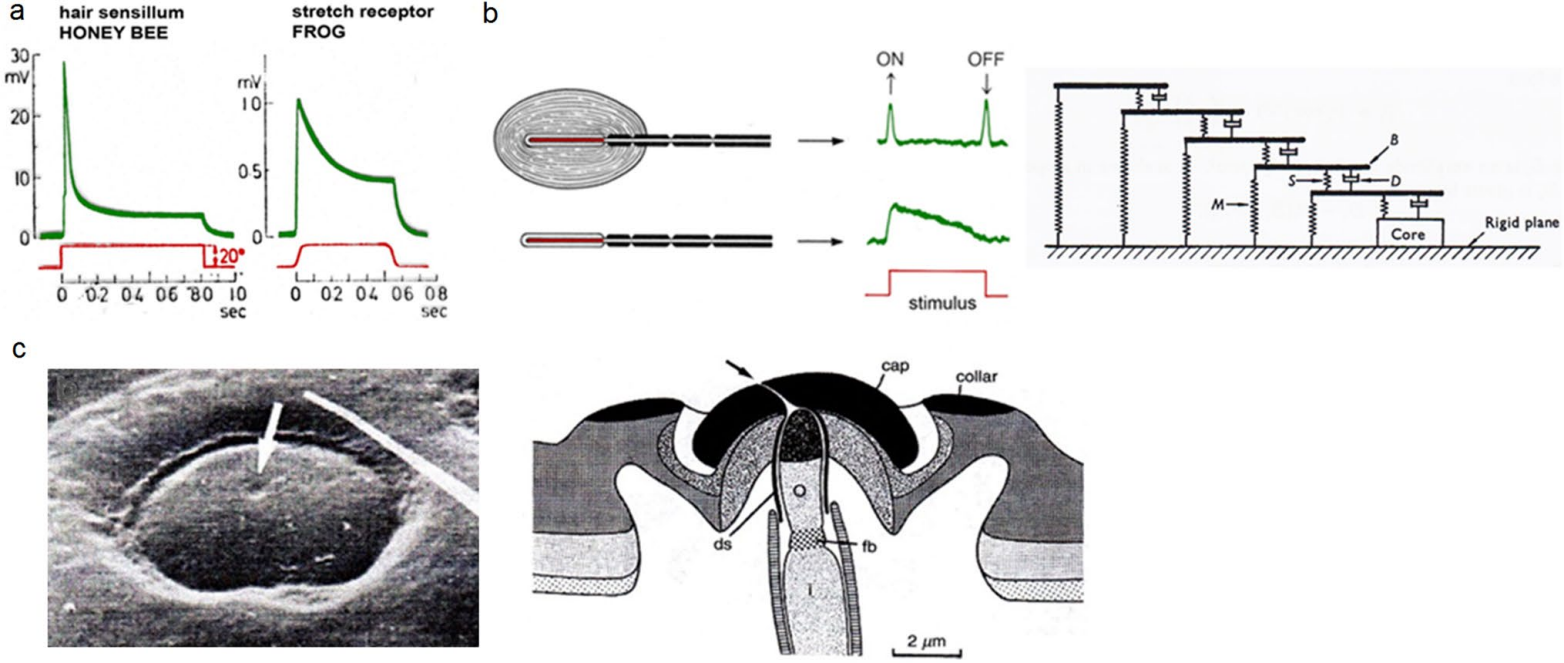
Viscoelasticity: **a** Receptor potential response of honey bee hair sensillum and frog stretch receptor to ramp-and-hold stimuli. **b** Vater–Pacini corpuscle (P.C., left) and its receptor potential in response to a tactile ramp-and-hold stimulus applied with the capsule intact (above) and the capsule largely removed (below). Right: Simple mechanical analog of capsule with elasticities (springs) and viscosities (dashpots) explaining stimulus transformation (high-pass filter). **c** Insect campaniform sensillum. Left: scanning electron micrograph of a sensillum (longer diameter c. 15 µm) on the fly (*Calliphora vicina*) leg. Right: schematic of section through stimulus conducting structures according to transmission electron micrograph and showing the different components with different shading. *ds* dendritic sheath, *fb* fibrillar body, *o* and *i* outer and inner segment of dendrite. (**a** Fuortes 1971, modified; **b** left: Loewenstein and Mendelson 1965, right: Loewenstein and Skalak 1966; **c** Grünert and Gnatzy 1987); with kind permission from Springer-Verlag GmbH (**a**, **c**) and Wiley and Sons (**b**)

The neural response of most mechanoreceptors to a ramp-and-hold stimulus declines with time (Fig. 2a). This decline during sustained stimulation needs not to be a decline in sensitivity as can be shown by the response to additional stimuli applied during the hold phase of the stimulus. It, often, is partly or completely due to the viscoelastic properties of the stimulus-transmitting non-neural structures. Thus, “adaptation” seen in the receptor potential or neuronal firing rate frequently in part or fully is not a neuronal but a non-nervous phenomenon. In any case, it helps to encode stimuli, which vary in time and the animals are mostly interested in. A few examples shall illustrate the impact of stimulus transmission due to such time-dependent coupling^1^.

The idea that viscoelasticity affects the response characteristics of mechanoreceptors has a history of well over 60 years (e.g., Loewenstein 1956, 1971; Hubbard 1958; Catton and Petoe 1966). It was applied to study the dependence of adaptation rate on stimulus velocity due to viscous coupling and to understand the dynamic and static phases of receptor responses (as, e.g., seen with ramp-and-hold stimulation). Frog and rat skin receptors, Pacinian corpuscles, slowly adapting muscle spindles, crayfish stretch receptors, and, later, other mechanoreceptors like insect campaniform sensilla, spider slit sense organs, and insect and arachnid medium-flow sensors (filiform hairs and trichobothria) were examined. In seminal early experiments, the response to mechanical stimulation was compared to that following direct electrical stimulation of the sensory cell. Muscle spindles (Lippold et al. 1960) and crayfish slow stretch receptors (Brown 1965) showed no decline of their response when directly stimulated electrically. Frog skin receptors (Catton 1966) responded after their typical latency only when stimulated mechanically. Doubtlessly, there is a fundamental effect of non-nervous stimulus conducting structures, although this is not to say that they are the only determinants. Even in the Pacinian corpuscle (see below) properties of the nerve terminal contribute to the adaptation of the response (Loewenstein and Mendelson 1965).

#### The Vater–Pacini corpuscles

As an introduction, let us start with a classic vertebrate case. The *Vater*–*Pacini corpuscles* are vibro-tactile mechanosensors deep in the mammalian skin, measuring about 1 mm by 2 mm (Fig. 2b). They have large receptive fields, which may comprise an entire human finger or much of the palm. Their nervous response to maintained stimuli is declining fast. They respond sensitively (skin indentation at threshold ca. 1 µm) to short-term contact and to vibratory stimuli with a best frequency of around 300 Hz.

Here, the structure of particular interest is what forms the major part of the “corpuscle”, the so-called capsule. It contains the receptive nerve ending and consists of 20–70 tissue layers arranged like the lamellae of an onion and separated from each other by viscous fluid. The stimulus has to pass through this onion and is dramatically modified by it on its way to the nerve ending. As elegantly shown in the 1960s already by Werner Loewenstein and his associates, the fast adapting response of the nerve terminal is largely explained by the viscoelastic properties of the capsule. Slow stimulus components simply do not reach the sensory ending. Upon the capsule’s removal the nerve ending does respond to maintained stimulation; its high-pass filter characteristic is gone and the receptor potential markedly prolonged. This is explained by a simple model consisting of springs (elasticities) and dashpots (viscosities) (Fig. 2b) (Loewenstein and Mendelson 1965; Loewenstein and Skalak 1966). Whereas the lamellae and their interconnections represent the elastic elements, the dashpots represent the lamellar surfaces and the interlamellar fluid.

According to more recent computer simulations by Quindlen et al. (2015), the deep placement in the skin helps the Pacinian corpuscle to detect stimuli over a large area of the skin, but increases the difficulty to identify their specific location. A refinement of the classical model of Pacinian corpuscle action by Loewenstein and Skalak (1966) is found in Biswas et al. (2014). These authors extended the model to a scalable one taking into account the size variability of the corpuscles, the material properties of the individual capsule layers, and their mass.

There is extensive literature on other mechanosensitive skin receptors, as well, relating firing rate of the nerve fiber to mechanical characteristics of stimulus (indentation force) transformation. An example is the slowly adapting type I afferents (SAI) of the Merkel cell complexes in the basal epidermal layer where skin viscoelastic relaxation was found to be a major contributor to firing rate adaptation upon stimulation by ramp-and-hold indentation (Williams et al. 2010). For the possible active role of epidermal Merkel cells in mechanosensory transduction, see a recent review article by Nakatani et al. (2015).

#### Arthropod strain sensors

##### Insects

Chapman et al. (1979) studied the role of viscoelastic coupling in sensory adaptation of campaniform sensilla. These are highly sensitive mechanoreceptors embedded in the insect exoskeleton and measuring cuticular strain (Fig. 2c). Though not quite applying the natural way of stimulation, these authors studied the time-dependent mechanical coupling of the stimulus by indenting the sensillum cap at various frequencies between 3 mHz and 100 Hz. Typically, the sensilla were rate-sensitive and stiffened with increasing frequency (some behaved purely elastically) and their discharge frequency increased with forcing frequency. The authors conclude that cap compliance exerts a mild filtering effect in case of the viscoelastic caps, but does not affect the decline of the nervous response (adaptation) in case of the purely elastic ones.

##### Spiders

**Fig. 3:**
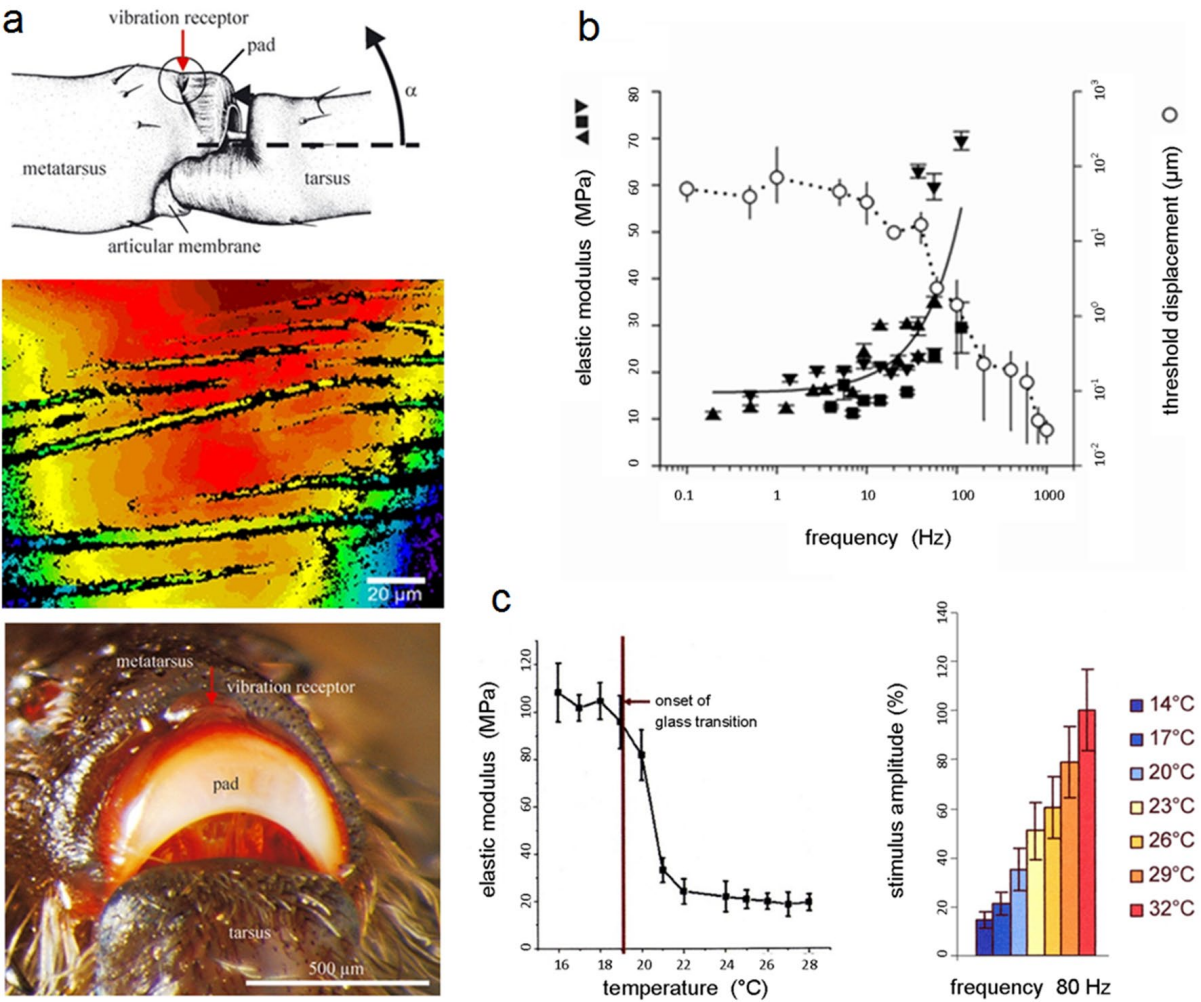
Viscoelasticity and cuticular strain receptors. **a** Top: Spider (*C.s*.) strain receptor serving as vibration receptor, located dorsally and distally on the metatarsus of the leg. Arrow indicates movement of the tarsus stimulating the organ by pushing (arrow head) onto the cuticular pad at the distal metatarsus. Middle: White-light interferometric picture of the slits composing the vibration receptor organ seen from above. Bottom: The distal end of the metatarsus showing the cuticular pad, which transmits the vibratory stimulus to the vibration receptor. **b** Frequency dependence of the pad’s elastic modulus (left y-axis) and a typical threshold curve of tarsal displacement (right y-axis) needed to elicit a slit’s nervous response (action potential) (*C.s*.). **c***Left*: The pad’s elastic modulus as a function of temperature showing the material’s glass transition range. Right: stimulus amplitude (tarsus deflection) needed to elicit a nervous threshold response of a slit at different temperatures (*C.s*.). (**a** Top: McConney et al. 2007; middle: Schaber et al. 2012; bottom: McConney et al. 2007; **b** McConney et al. 2007; **c** left: Young et al. 2014; right: Vogel 2008); with kind permission from the Royal Society London (**a**, **b**) and Elsevier Ltd. (**c**)

The intriguing power of viscoelasticity is also illustrated by the main spider vibration receptor, the so-called metatarsal organ. This is a compound or lyriform slit sense organ embedded in the exoskeleton on the most distal leg joint between metatarsus and tarsus and referred to as HS10 in the literature (Fig. 3a). Both its physiology and behavioral significance have been studied extensively (Barth 1997, 1998, 2002, 2012b). The metatarsal organ is stimulated by minute up and down (also sideways) movements of the tarsus induced by vibrations of the substrate. These play a significant role in courtship behavior, prey capture, and predator avoidance. The vibration receptor is stimulated, when the proximal end of the tarsus presses against the distal end of the metatarsus. The metatarsus, in turn, compresses and, thus, stimulates the innervated cuticular slits which make up the lyriform organ (Fig. 3a). Action potentials are then elicited and sent to the central nervous system. Viscoelasticity comes in when considering the pronounced high-pass characteristics of the physiological slit response: Threshold vibrations (in terms of displacement) eliciting a nervous response are high up to about 30 Hz, but steeply decrease at higher frequencies (Barth and Geethabali 1982). Apart from its high-pass property, the organ is not tuned to a particular frequency within the biologically relevant range. Irrelevant background noise (typically low frequency) is filtered out, and at the same time, high sensitivity is preserved for the higher frequencies typical of the biologically relevant vibrations such as actively produced courtship vibrations and vibrations produced by prey (Barth 2002). At the same time, the signal-to noise ratio is improved. As revealed by atomic force microscopy and surface force spectroscopy (McConney et al. 2007; Schaber et al.^2012^; Erko et al. 2015), the elastic modulus of the cuticular pad located in front of the metatarsal organ and transmitting the tarsal vibration to the vibration receptor strongly depends on frequency. It measures ca. 15 MPa at low frequencies up to ca.30 Hz, but 70 MPa at 112 Hz (Fig. 3a, b). The pad material has pronounced viscoelastic properties, with a glass transition temperature close to room temperature (25 ± 2 °C), and a maximized energy absorption at low-frequency vibrations. Importantly, the material properties suffice to largely explain the high-pass filtering seen in the nervous response. The spider may even make behavioral use of the pad material’s glass transition temperature (Fig. 3c, left), which lies in a biologically relevant range. *Cupiennius* is night active. It hunts prey when outside temperatures usually are lower by a couple of degrees as compared to daytime. The pad then becomes stiffer, transmitting the vibrations more effectively. The physiologically determined threshold sensitivity is indeed increased by such a temperature change (Fig. 3c, right) (Vogel 2008; Barth 2014). Remarkably, as shown by Young et al. (2014), the properties of the thick layer of epicuticle covering the pad alone explain the phenomenon.

Most of the so-called lyriform organs of spiders are found close to leg joints. Here, loads are introduced by muscular activity. They cause the strains in the cuticular exoskeleton, which stimulate the lyriform organs (Barth and Libera 1970; Barth and Stagl 1976; reviews: Barth 2002, 2012a, b). Analysis of the mechanical properties of the tibia-metatarsus joint of the spider *Cupiennius salei* showed viscoelasticity for lateral and dorsoventral loading. The strain induced in the distal tibia, where the lyriform organs are found, is, therefore, subjected to mechanical high-pass filtering. When comparing the electrophysiologically measured response of the slits of a lyriform organ (HS8) located distally on the tibia to stimulation by lateral ramp-and-hold deflections of the metatarsus with the force response of the joint, only a mild contribution of joint mechanics to the organ´s pronounced high-pass characteristics was found (Bohnenberger 1981; Blickhan 1986). Similarly, the time course of the decline of the response of campaniform sensilla of a cockroach to maintained stimulation is not primarily based on mechanics (Mann and Chapman 1975).

#### Arthropod medium-flow sensors

Viscoelastic properties have recently also been analyzed in some detail in spider medium-flow sensors (trichobothria) (McConney et al. 2009). The outstanding mechanical sensitivity of airflow sensory hairs to a large extent is due to the small elastic restoring force (*S*) at their suspension, which is on the order of 10^−12^ Nm rad^−1^. As revealed by force spectroscopic point load measurements and viscoelastic modeling, the suspensions of spider trichobothria have some interesting additional mechanical properties (McConney et al. 2009). The torque *T* (Nm) resisting hair motion increases steeply up to an angular velocity of ca. 0.06 rad^−s^. At about 6x10^−14^ Nm, the curve flattens drastically (Fig. 1b). The surprise was the very low torque values at very low angular velocities of hair deflection (below 0.05 rad^−s)^. In this range, a three-parameter solid model (two elasticities/springs in parallel, one dashpot in parallel) best describes the experimental data. For larger velocities, a two-parameter Kelvin solid model fits best (one elasticity/spring in parallel with one dashpot) (Fig. 1b). These viscoelastic properties of the hair suspension were suggested to promote the phasic response character of the hair response (Barth and Höller 1999; Barth 2002). At low frequencies, the dashpot can deform and the torque needed to attain a certain deflection decreases. The viscoelastic behavior of the material will facilitate the start of hair motion from rest and cope with the oscillating nature of hair deflection caused by and typical of the biologically relevant stimuli causing sudden changes of the hair’s angular velocity (McConney et al. 2009).

## Resonance and stochastic resonance

### For frequency tuning and selectivity

A parameter of foremost behavioral relevance contained in many mechanical stimuli is frequency content. Our ear is tuned to frequencies in our speech, many bats hear ultrasound used for echolocation in prey capture, grasshoppers listen to the sexual partner´s song, whales use infrasound to communicate over extremely long distances, and elephants do the same with infrasound vibrations introduced into the ground and received miles away. Medium-flow sensors and touch receptors are usually tuned to the relatively low frequencies (below 1 kHz) as they occur in air or water.

A difficulty to be mastered by all sensors is to pick up the biologically relevant signals and cues and to distinguish them from the ever present environmental noise. A flat response over a large range of frequencies usually is no solution. Instead, the receiver is often tuned to the frequency spectrum of the biologically relevant signals and thereby adapted to a particular animal´s life style and ecology.

Quite commonly sensors with their “best” frequency in the range of the biologically relevant signals and cues are qualified as “matched filters”, a terminology well-established in engineering. “Matched filters” represent correlation detectors, extracting the relevant stimuli from the environment and keeping out the non-essential ones from further processing, thereby also saving energy. This has already been emphasized for vertebrate auditory systems by Capranica and Moffat (1983) and Capranica and Rose (1983) and has recently been summarized by Narins and Clark (2016). Wehner significantly promoted the idea of sophisticated sensory filtering and highly specific selectivity in biology with an article entitled “Matched filters—neural models of the external world” (Wehner 1987). Warrant (2016) recently elegantly elaborated on the idea of sensory-matched filtering in biology (see also: Barth and Schmid 2001; von der Emde and Warrant 2016). Not to be forgotten is Jakob von Uexküll (1909), who more than a century ago as an early advocate of the idea stressed the uniqueness of the sensory worlds of different animals and the specificity of the signals their sensory systems have to cope with effectively.^2^

What can the non-nervous sensory periphery contribute to successfully deal with such problems?

#### Resonance for tuning and sensitivity

One way to adjust a sensor’s sensitivity to a particular frequency or range of frequencies is mechanical resonance. Resonance has to do with the free vibrations of an elastic body (oscillating system), which vibrates at its natural frequency, where energy absorption is greatest. If the frequency of forced vibrations (driven by an external force) equals the natural frequency, then we get “resonance”, where the vibration magnitude increases significantly. Since vibrational energy is stored, even small periodically driving forces may lead to large oscillations. With increasing damping of the elastic body and, thus, losses from cycle to cycle, the oscillation amplitude decreases and the range of resonant frequencies (peak of tuning curve) broadens (mostly defined by the Q factor, which describes bandwidth relative to center frequency and decreases with increasing damping; a high Q factor implies high selectivity of a sensor and a low rate of energy loss relative to stored energy). Some systems have several resonance frequencies, depending on the degrees of freedom.

Whereas engineers often have to take care to avoid resonance disasters by designing a bridge or an engine in a way that results in a mismatch between the natural and the driving frequency, resonance is used in sensory physiology to enhance sensitivity, to filter out biologically irrelevant frequencies, and to thereby improve the signal-to-noise ratio. A well-known example is the *meatus externus* of our human ear which is, by resonance, defined mainly by its length of 3.5–4 cm, amplifies frequencies between about 2 kHz and 4 kHz by up to 20 dB (“open-ear gain”). These frequencies are important in language communication (Ehret and Göpfert 2013).

##### Arthropod flow sensors

**Fig. 4:**
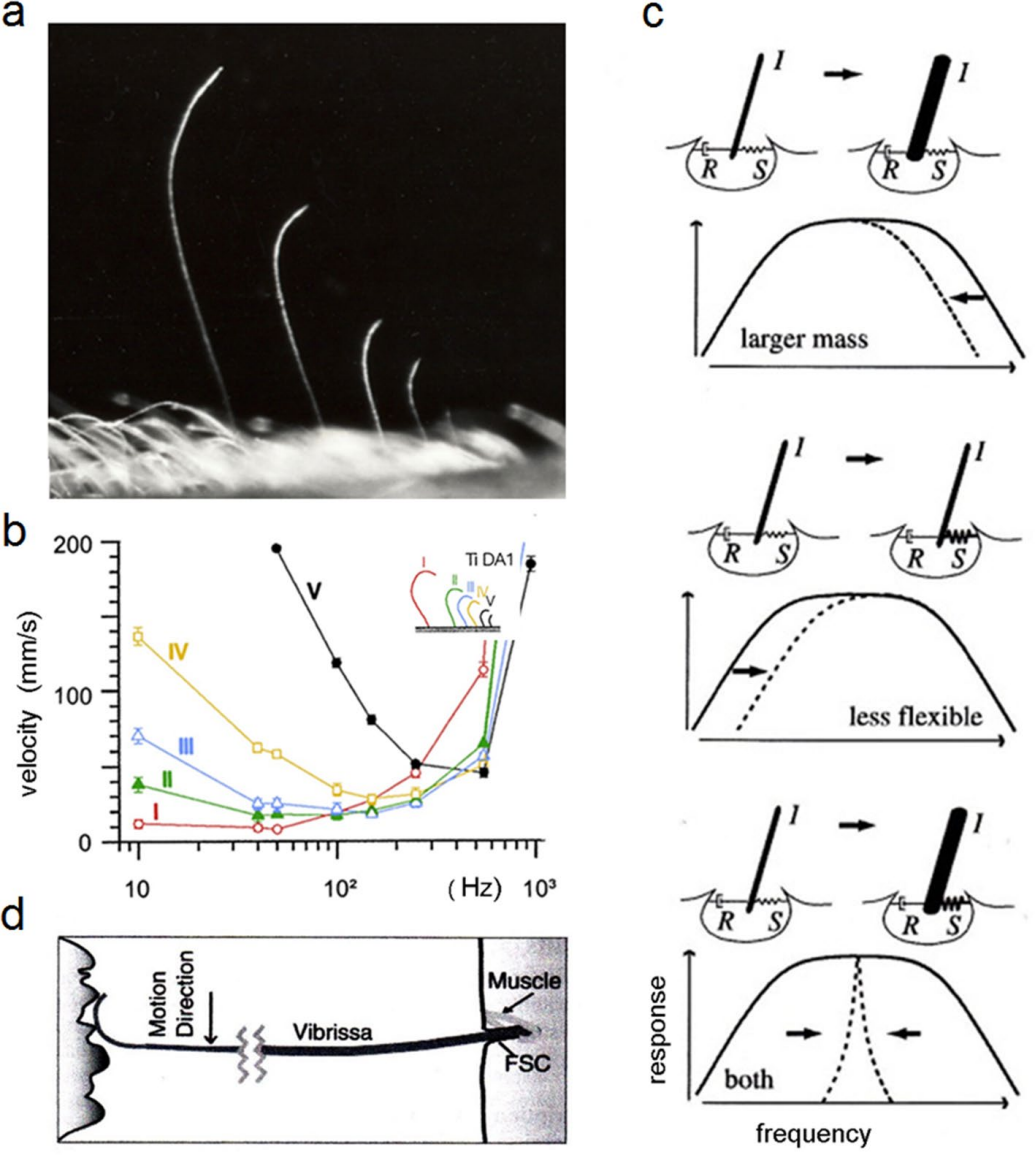
Resonance. **a** A group of spider (*C.s*.) trichobothria on the tarsus. **b** Mechanical frequency tuning of five trichobothria of different length on the spider (*C.s*.) tibia (length of hairs I–V 1150 µm, 700 µm, 650 µm, 500 µm, and 400 µm). Air-particle velocity necessary to deflect the hairs by 2.5° at frequencies between 10 Hz and 900 Hz. **c** Ways of mechanically modifying frequency tuning. Top: increasing the mass of the hairshaft decreases the upper boundary of the optimal frequency band; middle: increasing the elastic resistance of the suspension shifts the low-cat boundary of the optimal frequency range to higher values; bottom: hairs can become resonant by being less flexible and heavier. **d** Rat vibrissa sweeping past a surface with spatial frequency components causing it to deflect at specific frequencies. (**a** Barth unpubl.; **b** Barth et al. 1993, modified; **c** Bathellier et al. 2012; **d** Neimark et al. 2003); with kind permission from the Royal Society London (**c**) and the Society for Neuroscience (**d**)

Resonance also plays a role in the frequency tuning of hair-like medium-flow sensors (trichobothria, insect filiform hairs), which work like strongly damped inverted pendulums and mechanical band-pass filters (Shimozawa and Kanou 1984; Barth et al. 1993; Humphrey et al. 1993; Shimozawa et al. 1998; Shimozawa et al. 2003a, b; review: Humphrey and Barth 2008) (Fig. 4a). Both the maximum angular displacement and the maximum angular velocity resonance frequency of a filiform hair increase with increasing torsional restoring constant, *S* (Fig. 4c). The hair’s resonance frequency is also inversely proportional to hair length. Within a group of five hairs with pronounced length (*L*) gradation (*L* between 400 µm and 1150 µm), the frequencies eliciting greatest deflection varied between c. 600 Hz and 50 Hz (Fig. 4b) (Barth et al. 1993). Actually, the most effective way to change the mechanical frequency response of a filiform hair is by changing its length (Humphrey et al. 2003). Here, apart from changes in hair mass and suspension elasticity (going along with *L*), a match between hair length and boundary-layer thickness is an important aspect. Boundary-layer thickness generally decreases with increasing flow frequency. Hair resonance frequency increases with decreasing hair length. Interestingly, the frequency response of a hair correlates with the frequency of an oscillating flow where boundary-layer thickness scales with hair length. At resonance, the optimal hair length is not exceeding boundary-layer thickness (Humphrey and Barth 2008, p. 9).

##### Rodent whiskers

As analyzed by Neimark et al. (2003), rat whiskers resonate at and are finely tuned to frequencies between 50 Hz and 750 Hz depending on their length as well. They thus band-pass filter the incoming stimuli, facilitating their detection and recognition. Whereas the longer posterior vibrissae resonate at 60 Hz to 100 Hz, the short anterior ones resonate at c. 750 Hz. This is considered important for a proper neural representation of surface structure and may also allow for a decomposition of a stimulus into various components by parallel vibrissal channels. When whisking and generating oscillations of the vibrissae (Fig. 4d) and selectively amplifying high-frequency stimuli (Hartmann et al. 2003), rats are able to distinguish between finely grooved surfaces (depth in the µm range) and are thought to be able to deal with high-frequency information (Carvell and Simons 1990, 1995). The tuning is sharp, because the vibrissae are under-damped. The authors suggest that there may be a parallel between the resonance amplification and somatotopic encoding in vibrissal tactile processing and auditory encoding, considering cochlear resonances for the amplification of signals and the spatial map of frequency sensitivity (tonotopy) (Neimark et al. 2003).

##### Auditory hair cells—how to resonate at high frequencies?

Vertebrate hair cells like those in the inner ear carry a so-called “hair bundle” on their apical side, which consists of a dense array of stereovilli (often wrongly called stereo*cilia*). These are crucial in converting the incoming sound-induced mechanical vibrations to an electrical response of the cell membrane. They represent another case where passive elements affect stimulus transformation in a functionally highly significant way.

The tightly clustered hair bundle (size of gap between neighboring villi on the order of 1/10 of villus diameter only), mechanically connected to the transducer channels, represents a mechanical resonator which works/oscillates at frequencies up to more than 10 kHz. Considering the highly viscous endolymphatic fluid it is embedded in, this comes as a surprise, because the frictional forces and the much increased drag coefficient (as compared to an individual stereovillus) are expected to be high at Reynolds numbers much below one (on the order of 10^−4^). The hair bundle’s microstructure solves the mystery (Albert 2011; Kozlov et al. 2011). It forms an array of closely clustered stereovilli, which are connected and spaced by several linkers (*AL* ankle links, *HTC* horizontal top connectors, and *TL* tip links) along their length. As suggested by modeling and direct measurements (Kozlov et al. 2007, 2011), the fluid and all stereovilli move in unison in a sliding mode (as opposed to a squeezing mode), keeping their close apposition and gap width unchanged. Thereby fluid fluxes out of and into the bundle are largely prevented. The individual stereovilli are completely immersed in each other’s boundary layers. With the fluid inside the bundle largely immobilized and the fluid flowing largely around the array, the viscous drag associated with fluid flow and opposing the bundle’s deflection is much reduced. The hair bundle’s structure actually minimizes energy dissipation, making it easier for the active process (the cochlear amplifier) to keep the ear tuned (Kozlov et al. 2011).

##### Arthropod flow sensors

Medium-flow sensors of arthropods come in arrays as well (Fig. 4a). In groups of spider trichobothria, the distances between individual hairs were found to ensure a largely independent movement of individual hairs with practically no interaction for *s*/*d *> 50 (*s*, distance between hairs; *d*, hair diameter) and at most a very weak interaction for *s*/*d* > 20 and < 50 (the boundary layers of neighboring hairs not overlapping) (Humphrey et al. 1993; Bathellier et al. 2005). However, there is viscosity-mediated interaction between the more closely spaced and shorter filiform hairs on the cricket abdominal cerci (Humphrey and Barth 2008, p. 36; Cummins et al. 2007; Cummins and Gedeon 2012).

Active frequency filtering, which is well known of vertebrate ears, has now been shown for insect ears, as well (Göpfert et al. 2005; Römer 2016). It is not considered in the present review of passive mechanical events in the sensory periphery.

#### Stochastic resonance—maximizing sensitivity

A particular kind of resonance is the so-called stochastic resonance. It has been shown in several instances to play a role in the sensory measurements of weak stimulus intensities close to threshold by non-linear sensory systems. Here, sensitivity and information transfer are maximized by the presence of stochastic, uncorrelated input noise. It may seem counter-intuitive in the first place that the addition of noise, a usually undesired disturbance, might increase the probability of detecting a weak signal. The underlying principle was nicely made clear in an analysis of the action potentials in single mechanosensitive neuron innervating displacement sensitive hairs on the crayfish (*Procambarus clarkii*) tail fan (Douglass et al. 1993). By adding external noise to a weak periodic signal, the authors show noise-induced signal enhancement and the optimization of the signal-to-noise ratio at a certain average noise level and its decrease beyond this level.

Levin and Miller (1996) similarly stimulated airflow sensitive filiform hairs on the cercus of a cricket (*Acheta domestica*) and recorded the resulting action potentials from interneurons in the abdominal ganglion. They showed that stochastic resonance only works with signals of low intensity and at a particular level of noise, increasing information transfer by increasing the signal-to-noise ratio and the temporal coherence of the response with the stimulus. Other cases, where subthreshold stimuli are detected by the addition of noise are the noise-enhanced tactile sensations in humans (Collins et al. 1996), and also intensity detection thresholds in human hearing (Zeng et al. 2000). In the frog auditory system, on the other hand, stochastic resonance was not found, if the passing of the signal-to-noise ratio through a maximum with increasing noise level is taken as a criterion. However, increasing the internal noise level by changing the environmental temperature had a profound effect on the signal-to-noise ratio of stimulus encoding, increasing it (Narins et al. 1997). In a more general sense, then there is “noise benefits” as well (McDonnell and Abbott 2009; Wilkens and Moss 2008).

As mentioned above, spider and cricket airflow-sensing hairs are working close to k_B_T at threshold and will be exposed to the thermal noise of Brownian motion when working near threshold. This can be seen in the fluctuations of action potential timing (Shimozawa et al. 2003a, b). The intensity of external noise superimposed on a subthreshold sinusoidal signal and resulting in maximal enhancement (and a train of action potentials) corresponds to the receptor cell’s threshold. The enhancement of signal detection by coping with noise instead of avoiding it is again considered a case of stochastic resonance (Shimozawa et al. 2003a, b)^3^.

## Traveling waves

### For spectral analysis

#### Vertebrate cochlea

Traveling waves have been a fascinating topic in hearing physiology since their first discovery in the vertebrate inner ear by Nobel laureate Georg von Békésy (von Békésy 1960). Their sensory relevance may shortly be described as tonotopy, which means place-based frequency discrimination. Von Békésy built models of the cochlea and observed undulating waves slowly traveling along the model basilar membrane when he set the cochlear fluid into motion. By adjusting the tension of the membrane, he could confine the biggest part of the observed bulge to a particular region along the length of the membrane. He observed the same type of wave in animal (mammals from mouse to elephant) and human ears. His place theory states that a sound impulse initiates a traveling wave, whose amplitude increases until it reaches a maximum and then falls off sharply, the high frequencies being represented near the base and the low frequencies near the apex of the cochlea (passive tuning). As is well known by now, the cochlea is a spectral analyzer. Its basilar membrane responds to the pressure changes received from the middle ear with largely independent traveling waves (surface waves—smaller amplitudes, slower propagation velocity than sound in liquid medium: wavelengths on the order of 1 mm, displacement < 1 µm even for loud sounds) for each frequency component. Because of graded mass and stiffness along the membrane, the traveling wave grows in magnitude and decreases in wavelength until it reaches its peak amplitude at its frequency-specific location. There, the hair cells are not only activated regarding their nervous response (depolarization). They also actively add to the energy of the membrane´s movements by counteracting viscous damping. They thereby improve frequency discrimination and the tuned mechanical amplification of weak signals. Thus, the sharp frequency selectivity of the cochlea is not only based on the sound decomposition by the traveling wave (frequency-to-place transformation) and the sharp decline apical to its peak amplitude. Actually, the traveling wave is, to a large extent, an epiphenomenon, each region of the organ of Corti essentially responding independently of its neighbors and with its own characteristic delay (Manley 2018). The energy generating the traveling wave comes from the considerably faster pressure wave, which impacts the entire basilar membrane within a few microseconds only. Thus, the traveling wave is the effect rather than the cause of the different local displacements of the organ of Corti (Manley 2018). It is based on local resonances along the membrane changing with mass and stiffness, basically as suggested more than a century ago by von Helmholtz (1885). From base to apex, stiffness decreases and mass increases exponentially; the resonant frequency follows an exponential map from high frequencies near the base to low frequencies near the apex; thereby, the large frequency range it responds to (logarithmic arrangement of frequency) (see recent review by Reichenbach and Hudspeth 2014).

The estimated power dissipation of the cochlea for the frequency–to-place transformation of sound and the compression of an extremely large input dynamic range of 120 dB (at 3 kHz) into a 30–40 dB output range in nerve firing rate is only 14 µW, not to forget that in its most sensitive frequency range sub-Ångström displacements of the ear drum are sensed (Johnstone 1967; Sarpeshkar et al. 1998; Sarpeshkar 2003).

#### Insect ears

##### Abdominal ears

**Fig. 5:**
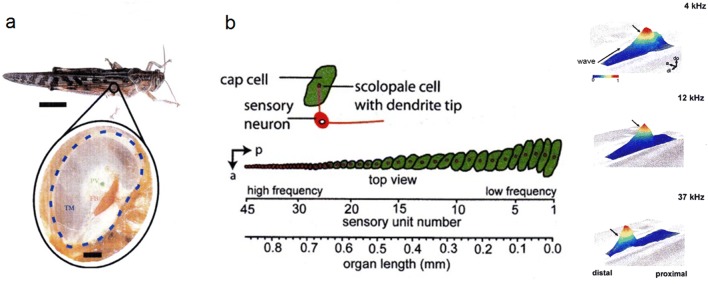
Traveling waves. **a** Tympanum of the abdominal locust ear (*Schistocerca gregaria*). Scale bars: body 12 mm, membrane 0.2 mm; *TM* tympanal membrane, *PV* pyriform vesicle, *FB* folded body. **b** Left: Crista acustica of the hearing organ of a katydid (*Mecopoda elongata*) on its front leg tibia; cellular elements of the sensory units (scolopidia) involved in the transformation and transduction of the acoustic stimuli. *a* anterior, *p* posterior. *Right*: Examples of normalized waves induced by sound of different frequency; note tonotopy. (**a** Windmill et al. 2008; **b** Hummel et al. 2016); with kind permission from the Royal Society London (**a**) and the Society for Neuroscience (**b**)

The virtues of traveling waves also follow from their presence in *insect hearing* where they must have evolved independently from the mammals and other vertebrates like amphibians (Hillery and Narins 1984). In the desert locust, a traveling wave of the anisotropic eardrum (tympanum) is responsible for the spatial decomposition of sound into frequency components and its tonotopic representation (Fig. 5a) (Windmill et al. 2005). Locusts have their eardrums on each side of the first segment of their abdomen. The wave always travels from the thin membrane area of the tympanum to the thick area of the membrane. The sound energy is funneled to specific locations where groups of mechanoreceptor units (four clusters of the multicellular scolopidia making up Müller´s organ) are located. These have been known to respond best to different frequencies for a while already, the membrane mechanics being responsible for different resonance frequencies in different areas (Michelsen 1968, 1971a, b; Römer 1976). In this case then, the structure receiving sound and decomposing it is the same, different from the basilar membrane in the mammalian cochlea. The velocity of the wave is between 9.6 and 15.9 m/s at frequencies up to 30 kHz and < 10 kHz, respectively. These values are similar to those found in mammalian cochleae, despite the differences in geometry and length scales.

Similar signal partitioning by thin and light eardrums which can be easily set in vibrations (amplitudes in the nanometer range) by sound pressure is, in addition, found in *cicadas*. These have their eardrums on both sides ventrally in the second abdominal segment. Again, frequency-specific traveling waves are observed in a heterogeneous tympanal membrane (*Cicadatra atra*). The deflection shapes of this membrane are similar to those known from the mammalian basilar membrane. There are frequency-dependent locations of amplitude maxima along the dark “tympanal ridge” (length c. 1 mm), which crosses part of the tympanum and links it with an apodeme to the receptor cells. Clearly, these vibrations differ greatly from what would be the resonant modes of a homogeneous drum skin (Sueur et al. 2006).

##### Tibial ears

The relevance of the above findings is still enhanced by yet another “grasshopper”, an Asian tropical bushcricket (*Mecopoda elongata*, Tettigoniidae), which has its hearing organs in the tibiae of the front legs (Udayashankar et al. 2012; Hummel et al. 2016). Different from the abdominal locust ear, some 45 distinct mechanosensory units (scolopidia, containing the dendrite of a bipolar sensory cell, and a few supporting cells including a scolopale cell and a cap cell) are arranged in a *crista acustica*. The *crista* is about 1 mm long (Fig. 5b). It lines the dorsal side of the acoustic trachea, which is shaped like an exponential horn, works as a high-pass filter and amplifies by up to 20 dB (Hoffmann and Jatho 1995). Measurements of the mechanical tuning at different locations along the anatomically graded crista again show tonotopy, with low frequencies represented by the broad proximal part and the high frequencies setting the narrower and stiffer distal part in motion. It is mainly the gradient in cap cell size which provides a mass gradient along the crista (Fig. 5b). The velocity of the traveling wave and its wavelength depend on the local mechanical properties at a specific location of the crista. Similar to the mammalian case, the build- up of the peak displacement is gradual, whereas the subsequent decline is much faster.

According to electrophysiological recordings from the neurons associated with the different sites of motion peaks of the crista, the action potential activity at any frequency is largest during the ascending phase of the wave motion. The mechanical tuning curves are broader than the neural tuning curves. The phase delay leads to a tilt between the cap cell and the dendrite tip, which is thought to induce the opening of mechanosensitive membrane channels (Udayashankar et al. 2012; Hummel et al. 2016).

## Directional selectivity

### To know where the stimulus comes from

Animals are in constant search of energy. This is a consequence of thermodynamic law and the fact that energy-wise they are all open systems. Most likely, this is one of the main reasons for their outstanding sensory capacities. Where is the prey? Where is the mate? Where is the predator? These are among the crucial questions, on which the survival of the individual and the evolutionary success of a population and species depend. Accordingly, the directionality of sensors is an issue of high biological relevance. Often, it is achieved by the mechanical properties of structures involved in stimulus uptake. Examples are the following.

#### Arthropod hair sensilla

**Fig. 6:**
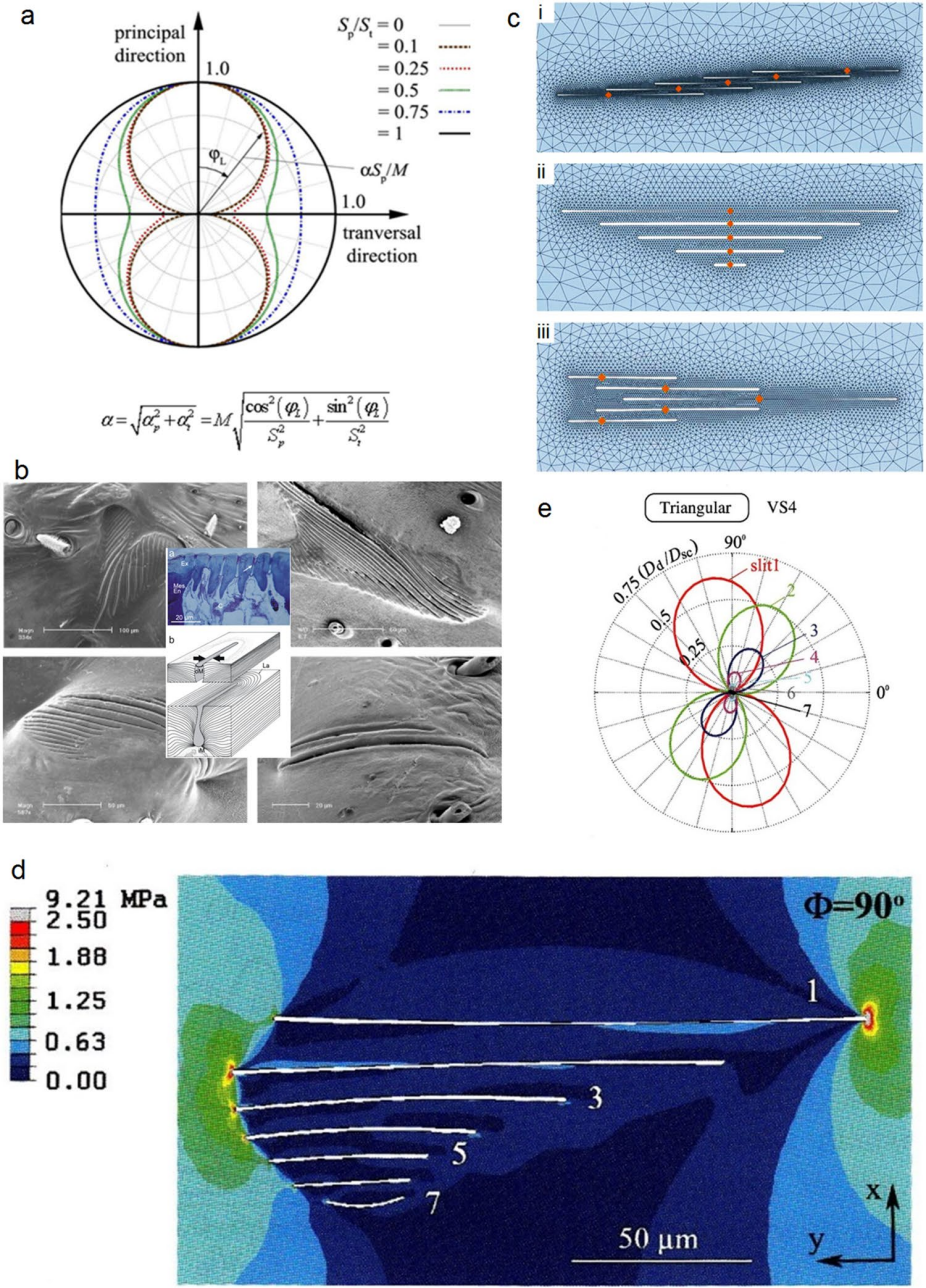
Directionality. **a** Directional characteristics of the joints of arthropod hair-like sensilla; polar plot derived from mathematical model for different ratios of joint stiffnesses in the preferred (*S*_*p*_) direction and the direction transversal to it (*S*_t_). *ϕ*_L_ load direction, *α* deflection angle, ϕ_α_ direction of deflection, and *M* moment introduced to joint. **b** Lyriform slit sense organs of different outline shapes on the spider (*C.s.*) leg. *Center*: Histological cross section through slits and arrangement of cuticular laminae (*La*) forming it; arrows indicate adequate stimulation of the slit by its compression. *oM* outer membrane covering the slit, *iM* inner membrane, *Ex,Mes,En* exo-, meso-, and endocuticle, *c* cellular components. **c** Deformed configurations of three FE models of lyriform slit sense organs subjected to uniaxial compressive loading at an angle of 90° with regard to the slit long axes. **d** Von Mises equivalent stresses (MPa) at model slits arranged as in a specific natural lyriform organ (VS4) of the spider *C.s*. Load at 90° to slit long axes. **e**. Directional mechanical sensitivity (given as *D*_d_*/D*_sc_, the ratio between slit face deformation *D*_*d*_ at the dendrite’s position and the displacement *D*_sc_ at mid-length of a single isolated slit) of the organ shown in **d** under uniaxial compressive far-field loads from different directions. Note remarkable differences between the slits and the big changes of slit face deformation even with small changes of load direction. (**a** Dechant et al. 2006; Barth 2016; **b** Barth 2012a, b; Hößl et al. 2009; **c** Hößl et al. 2007; **d**, **e** Hößl et al. 2009); with kind permission from Springer-Verlag GmbH (**a**–**e**)

In mechanosensitive arthropod hair sensilla, the type and degree of directionality of hair shaft deflection largely depends on the presence or absence of limitations due to a directional structure of the stiff cuticular socket (e.g. forming a mechanical stop in certain directions and not in others) and/or the degree of homo- or heterogeneity of the hair’s membranous suspension (e.g., Gnatzy and Tautz 1980; Dechant et al. 2006; see also Barth 2016). The directional dependence of the torque resisting the deflection of tactile hairs is readily measured. Thus, for tactile hairs at a joint of the spider leg, the elastic restoring force opposing hair deflection was found to be smaller by about two powers of ten in the direction of natural stimulation (c. 5x 10^−12^ Nm deg^−1^) as compared to all other directions (Schaber and Barth 2015). Dechant et al. (2006) provide a simple mechanical model of directionality, which can be applied to any anisotropic hair suspension reacting with different stiffness to loads applied from different directions (Fig. 6a). The mechanical directionality can be quantitatively described when knowing (1) the joint stiffness in the preferred direction (*S*_p_) and in a plane transversal to it (*S*_t_) and also (2) the stiffnesses for the opposite directions^4^.

#### Vertebrate hair cells

The stereovilli (microvilli densely filled with actin filaments) on the apical surface of vertebrate hair cells are rigid rods graded in length, which increases in a staircase manner towards the longest villi and sometimes a kinocilium (which is a true cilium). Deflection of the bundle towards the tall edge entails a gliding movement between the stereovilli and causes the opening of mechanosensitive channels, whereas deflection in the opposing direction closes them. This goes along with excitatory depolarization and inhibitory hyperpolarization of the cell membrane, respectively (Flock 1965; Wersäll and Flock 1965; Hudspeth 1985). The tip links and additional links between neighboring stereovilli are crucial in explaining this phenomenon: Only in the first case, they are stretched by the applied force; tip link loss eliminates mechanotransduction (Assad et al. 1991; Zhao et al. 1996; review: Peng et al. 2011).

#### Arthropod strain detectors

##### Spider slit sensilla

In arthropod strain sensors like spider slit sensilla (Barth 2012a, b), which are embedded in the exoskeletal cuticle, directionality is due to structure and stimulus transmission, as well. Slit sensilla may, indeed, be the most complex and refined example of directionality due to non-nervous structures in the sensory periphery. They form narrow (width 1 µm to 2 µm) elongated slits with a thin (0.25 µm) covering membrane to which one of two sensory cells attaches (Fig. 6b) (the other one ending near the membrane closing the slit on its inside; Barth 1971). Being sites of increased compliance and dealing with the bone-like stiffness of the surrounding cuticle, the slits are stimulated by the slightest compression down to the nanometer range (at threshold down to c. 1.5 nm) and resulting from cuticular strains as small as − 10 to − 20 µɛ (− 2 × 10^−5^) (Blickhan and Barth 1985). The slits vary in length from c. 8 µm to c. 200 µm, and their aspect ratios are between c. 10 to100 (Barth and Libera 1970). In simplified form, one can interpret the borders of single slits as bending beams. They will increasingly bend the longer the slit is and the more the stimulus direction is oriented at right angle to the slit long axis (Barth et al. 1984). Thus, slit compression greatly depends on stimulus direction.

The most conspicuous slit sensilla are the so-called *lyriform organs*. They form arrays of up to 30 slits closely arranged in parallel (interslit distance about 5 µm). Their majority is found close to leg joints (review: Barth 2012a, b, c) (Fig. 6b). When exposed to compressive strains, the mechanical interaction of several slits of similar or different length so closely arranged in arrays affects both the absolute and directional mechanical sensitivity of a slit in a complex way. Much depends on a slit´s more peripheral or central location within the group. These mechanical effects were analyzed in some detail, first using tension optical techniques (Barth et al. 1984), later Finite-Element Modeling (Hößl et al. 2006, 2007, 2009), and finally white-light interferometry for studies of intact natural organs (Schaber et al. 2012). The different arrangements of slits in lyriform organs (represented by groups of five slits in the models) (Fig. 6c) vary strongly regarding the distribution and degree of mechanical sensitivity and directionality within the group. They are, therefore, suggested to be pre-disposed for physiological functions vastly differing on structural grounds and mechanical “pre-processing” (filtering) alone.

To be more specific, the main influential parameters are lateral spacing, longitudinal shift between the slits and their length gradation. Slit face displacement varies a lot with these parameters. The effects include both mechanical amplification (compared to a single slit) and shielding and also determine the directional sensitivity of the slits (review: Barth 2012a, b). In a non-staggered array with spacing as close as in natural organs (S/l_o_ ≤ 0.1; S, lateral spacing; l_o_, slit length), the inner slits are strongly shielded by the outer slits and, therefore, their compression is much reduced. Lateral shift *λ* of equally long slits in an oblique bar arrangement may amplify deformation of the slits by more than 400% as compared to that of a single slit, provided 0.25 ≤ *λ*/l_o_ ≤ 2.5. In a “triangular” array of slits (Fig. 6c, d) (referring to the outline of the lyriform organ) differing in length, one gets widely differing responses of the slits, presumably much enlarging the working range regarding signal magnitude in the original organ. By arranging the slits in a way resulting in a heart-shaped outline, the angular range of sensitivity can be significantly enlarged. This also applies to a non-parallel, fan-like arrangement of slits.

Thus, the mechanical properties of diverse slit arrangements suggest mechanical pre-processing by being adapted to different functional properties such as having a particularly large working range, responding to strains in a small or large angular range or, as in case of slits of equal length arranged with constant lateral shift, increasing the signal-to-noise ratio by the central nervous convergence of the identical responses of several slits. When analyzing models even closer to the natural geometries (in regard to number of slits, lateral and longitudinal shift, curvature, and angle between slits), even minor changes were found to potentially greatly change the directional sensitivity of a slit (Fig. 6e) (Hößl et al. 2009).

Finally, the predictions drawn from the FE simulations could be valued and supported by a comparison with data gained for an intact natural lyriform organ (the “triangular” lyriform organ HS-8) previously studied most extensively (electrophysiology: Barth and Bohnenberger 1978; Bohnenberger 1981; on site strain measurements under natural behavioral conditions: Blickhan and Barth 1985; Brüssel 1987; interferometric measurement of slit deformation under controlled load conditions: Schaber et al. 2012).

##### Insect campaniform sensilla

Insects have strain sensors embedded in their exoskeleton as well, known as campaniform sensilla and analogous to the arachnid slit sensilla (Pringle 1938; Moran et al. 1971; Gnatzy et al. 1987; Grünert and Gnatzy 1987; Barth 2012a). They form holes in the cuticle as well, with a dome-shaped cuticular cap covering them on the outside and the dendrite of a sensory cell attached to it (Fig. 2c) (Grünert and Gnatzy 1987). Although the aspect ratios of slit sensilla with values up to 100 and more exceed those of campaniform sensilla by far, the majority of these is not round but oval, one axis being longer than the other (e.g., Gnatzy et al. 1987). As expected, such campaniform sensilla are directionally sensitive, responding most strongly to compressive strains perpendicular to their long axis (including that of the cuticular collar surrounding the cap), just like the slit sensilla. A particularly well-studied case is the 9–14 campaniform sensilla on the proximal tibia of the cockroach. They come in two subgroups with their long axes oriented perpendicular to each other (parallel and perpendicular to leg long axis, respectively) (Zill and Moran 1981; for the stick insect see: Zill et al. 2013). Interestingly, by computational modeling, aspect ratio was found to be of little influence on the opening deformation of holes for values between c. 20 and 100, which are typical of spider slit sensilla. It is very relevant for the round (aspect ratio 1) to elliptical campaniform sensilla, however, where aspect ratios are mostly below three (Hößl et al. 2007). The reader is also referred to another elegant and more recent study on the trochanteral groups of campaniform sensilla of a stick insect and their nervous response when loaded by self-generated or imposed forces (strains) naturally occurring in the exoskeleton (Zill et al. 2012).

## The cellular and molecular level

### Gating the channels for a fast response

In the end, in all mechanoreceptors, an adequate stimulus changes the permeability of the mechanosensory cell by changing the open probability (step responses) of mechanosensitive membrane channels (MSC) due to membrane tension. Again, we are dealing with questions of mechanics and micromechanical measurements are instrumental in an effort to find out what the mechanisms are behind these final steps. As is well known, mechanoreceptors excel in regard to the short latency of their responses, which may be in the sub-millisecond range and often measures only microseconds, such as only c. 40 µs in bullfrog hair cells (Corey and Hudspeth 1979; Thurm et al. 1983; Albert et al. 2007). The general assumption, therefore, is that the mechanosensitive ion channels are gated by the stimulus force directly. This differs from senses like vision and chemoreception, which rely on second messengers, biochemical cascades, and diffusion processes taking much longer. However, “directly” does not mean that stimulus transformation has stopped here. On the contrary, in its final stages, stimulus transformation is still complex.

#### Hair cells

Despite a lot of fascinating research, there is still debate about the final steps of stimulus uptake and transformation in hair cells. Inner ear hair cells have been a focus of mechanotransduction research for a long time. According to the gating spring model tip links between neighboring stereovilli function analogous to strain gauges when the hair bundle is deflected. They respond to tension by pulling on a spring and thereby control the load (force) on the channels and their conductivity. The load is expected to increase with the stiffness of the spring/elastic element. Details of the mechanics of this process, which implies matching the difference in mechanical impedance (see below) between the hair bundle and the transduction channels, are still not clear enough (Hudspeth 2014). Thus, the function of the spring may reside in the tip link, the cell membrane, or an unidentified linker inside the sensory cell (Powers et al. 2012; Zhao and Muller 2015). Another goal of research into hair cell mechanotransduction remains the identification of the channels proper, that is the pore-forming proteins (Zhao and Muller 2015). The stiffness of the spring is in the range of pN/nm. It provides the system with a compliance probably taking up a substantial part of the incoming deformation and matching it to the much smaller nm-conformational changes of the transduction channels. The original interpretation of the tip links as the compliant gating springs in vertebrate hair cells has later been questioned, because their stiffness is much larger than expected for the gating spring (Gillespie and Muller 2009). Both absolute sensitivity and dynamic range will depend on the magnitude of this compliance. A comparative study of the mechanical coupling and, thus, the gating of the transduction channels and its functional consequences in different mechanoreceptors would certainly be rewarding.

#### Arthropod “hair” sensilla and strain sensors

##### Hair sensilla

**Fig. 7:**
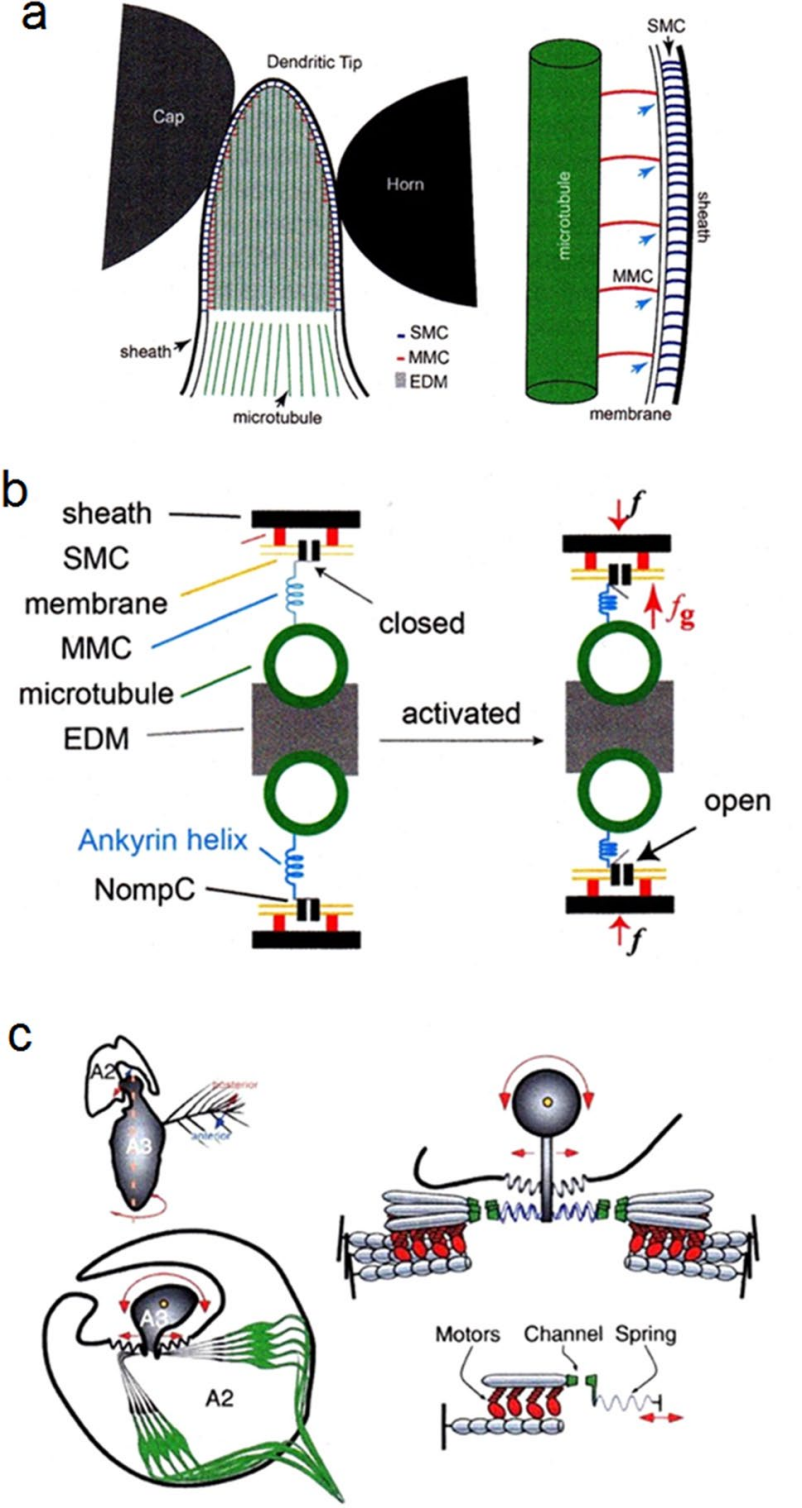
The cellular and molecular level. **a** Dendritic tip and supporting structures of *Drosophila* hair-like sensilla. *SMC* sheath-membrane connector; *MMC* membrane-microtubule connector; *EDM* electron-dense material. **b** Linkage model of mechanotransduction proposed for campaniform sensilla and possibly also applicable to mechanosensitive arthropod hair-like sensilla. External force (*f*) is transformed to gating force (*f*_g_) opening the mechanosensitive transduction channel. The gating spring may be the Ankyrin helix **c** Transducer-based model oft he *Drosophila* ear. *A* Front view of antenna showing the two distal segments A2 and A3, the latter carrying the feathery arista. The arista together with A3 takes up the sound signal, being moved back and forth by frictional forces. Bottom: cross section of joint between A2 and A3. Arrows indicate movements evoked by sound stimulation. Note two populations of mechanosensitive sensory cells (green). *B* Model showing two opposing transducer modules (consisting of one ion channel, a set of adaptation motors and one gating spring) coupled symmetrically to a harmonic oscillator. (**a**, **b** Liang et al. 2017; **c** Nadrpowski et al. 2008); with kind permission from Springer-Verlag GmbH (**a**, **b**) and Elsevier Ltd. (**c**)

The site of transduction in hair sensilla is where the dendritic tips are coupled to the inner arm of the (first-order) levers (Fig. 1a, d). In *Drosophila,* NOMPC/TRPN mechanotransduction channels have been found only there (Liang et al. 2011). As has been known for a while already (Thurm et al. 1983), the ultrastructure of the dendritic tip region is quite elaborate (Fig. 7a). From outside in, there is an extracellular dendritic sheath, which is connected to the cell membrane by sheath-membrane connectors (SMCs). The inside of the dendritic tip of cuticular arthropod mechanosensory cells typically shows a so-called tubular body, consisting of microtubules embedded in electron-dense material. The peripheral microtubules are connected to the cell membrane by membrane-microtubule connectors (MMCs). These structural specializations at the dendrite tip represent the final mechanical pathway to the transduction channels proper. These are believed to be NOMPC/TRPN channels gated by membrane strain due to compressive forces induced by hair deflection (Liang et al. 2011). MMCs are suggested to focus the stimulus onto restricted membrane areas (Fig. 7b). Their individual stiffness was estimated to be on the order of 3 pN/nm already 35 years ago (Thurm et al. 1983). Interestingly, spider mechanosensitive hair sensilla (at least the several types so far studied) differ from the insect ones (Keil 1997a, b) by not being directly coupled to the inner arm of the lever. Instead, they are separated from it by a strand of material looking homogeneous in the transmission electron microscope (Barth et al. 2004; Barth 2016). The mechanical properties of this material are unknown but most likely significant regarding stimulus transmission. The functional significance of this difference between insects and spiders is not clear yet. Corresponding nano-mechanics still waits to be done.

##### Campaniform sensilla

As mentioned before, these are insect strain sensors embedded in the cuticular exoskeleton and supplied by a single bipolar sensory cell. The ultrastructure of the dendritic tip region is similar to that of the hair sensilla as described above (Grünert and Gnatzy 1987). It shows a dilated tubular body and a flattened tip (oval cross section) where the stimulus is presumed to be taken up by the sensory cell. Campaniform sensilla are particularly sensitive to stimuli perpendicular to the long axis of the dendritic tip (see above). The microtubuli are arranged in two rows, interconnected by electron-dense material, and linked to the cell membrane by MMCs (membrane-microtubule connectors). It is here that the transduction proper is supposed to be localized, the exclusive occurrence of NOMPC in this region being a strong argument (Liang et al. 2011, 2013). Like in the hair sensilla, SMCs connect the dendritic sheath to the cell membrane.

The mechanical model currently offered for the function of the final steps in stimulus transformation and the channels’ gating by force is summarized in Fig. 7c (Liang et al. 2017). The respective role of compressive forces, with the channels being tethered to extracellular matrix or intracellular cytoskeleton, or lateral membrane tension still is not clear (Zanini and Göpfert 2013). A good molecular candidate for the gating spring of NOMPC channels seems to be their ankyrin repeats (Jin et al. 2017). According to Zhang et al. (2015), the NOMPC ankyrin spring is part of the MMCs.

#### Johnston organ of Drosophila

The antennal insect “ear” looks very different from the vertebrate ear and indeed from the tympanal ears of insects as well. Similar to the spider trichobothria and insect filiform hairs, it responds to the movement (particle velocity) instead of the pressure aspect of the acoustic stimulus as the tympanal organs do. It, therefore, is a near-field receptor preferably responding to low frequencies below 1 kHz. The Johnston organ is driven by frictional forces exerted by moving air. Despite these differences to the vertebrate ear, there are striking similarities, like the very short latency and the underlying direct mechanical coupling of the stimulus to the sensory cell membrane containing the mechanosensitive ion channels. The following mainly draws from the work on *Drosophila* summarized in Albert et al. (2007) and Albert and Kozlov (2016).

The primary sound receiver is the so-called *arista*, a unilateral feathery appendage on the third antennal segment (funiculus*)* (Fig. 7c). When exposed to sound, the funiculus together with its arista rotates about its longitudinal axis, working like a damped harmonic oscillator with a linear response behavior (Göpfert et al. 2005). Its vibrations alternately stretch and compress two populations of sound-sensitive sensory neurons in Johnston’s organ, which is located in the second antennal segment, the pedicellus (Fig. 7c). The modules coupling the stimulus to the ion channels make the entire insect ear a non-linear and also an actively amplifying system (Göpfert et al. 2005).

Remarkably, when applying force steps (c.− 100 pN to + 100 pN) to the arista and measuring the displacement of its tip (≤ 3 µm), similarities to the results of analogous experiments with vertebrate hair cell hair bundles show up: an initial overshoot reflecting the channel opening and a subsequent adaptation to a steady state (constant offset), following the gating spring model and indicating elasticities connecting to the channel gate. Sub-millisecond response latencies between the force step moving the arista and the nervous response (compound action potential) reflect and confirm the direct channel (mechanotransducer) gating by gating springs (Fig. 7c) (Albert et al. 2007).

## A photomechanic mechanism

### For an insect infrared receptor

There is a bizarre beetle closely associated with forest fires like some other 25 pyrophilous insect species (Schmitz and Bleckmann 1998; Schmitz et al. 2016). The larvae of *Melanophila acuminata* (Buprestidae) depend on wood from fresh fire-killed trees. Adults fly towards forest fires, and copulate and lay eggs under the bark of burnt trees as soon as the flames have subsided. Obviously, the beetles are attracted from large distances of many kilometers (Evans 1964). They have about 70 closely packed dome-shaped cuticular infrared (IR) receptors lying in each of two sensory pits on their metathorax. Each IR receptor contains a cuticular sphere (diameter c. 10 µm) with the dendritic ending of a mechanosensory cell on its inner side. According to present knowledge, IR radiation absorbed by the sphere is thought to increase the temperature and as a consequence to increase the pressure inside the sphere, squeezing the dendritic tip of the mechanoreceptor cell. For a recent review of infrared reception and an extensive list of literature, see Schmitz et al. (2016).

## The complex question of impedance matching

### To increase sensitivity in ears, filiform hairs, and the elephant’s vibration sense

Impedance matching may be best known from electrical engineering where the input impedance of an electrical load or the output impedance of the relevant signal source has to be designed in a way that allows maximal power transfer. The older generation among the biologist readers will remember the times when cathode followers were a big issue in electrophysiology (Whitfield 1960). When recording neuronal activity, it was important to adjust the high output resistance of the voltage source (the sensory cell or nerve fiber) to the low input stage resistance of the amplifier (oscilloscope) to avoid loss of gain (voltage) or signal distortions.

Impedance matching refers to the transfer of other forms of energy as well and actually what was reviewed so far already had a lot to do with it. To transfer as much energy as possible from one stage of stimulus transformation to the next is an issue relevant for all sensory systems and stimulus modalities. A classical example for the significance of impedance matching in a sensory system is the transfer of acoustic energy from one medium to another, like from the surrounding air to our human fluid-filled inner ear. Without impedance matching, most of the energy would be reflected and not enter the cochlea. Here, only a few examples are given to further stress the general significance of impedance matching. Judging from the low physiological thresholds close to background noise found in so many cases, one may conclude that the overall impedance matching comprising several steps of transfer is quite efficient.

#### The human ear

Impedance matching between airborne sound meeting the human ear and the subsequent chain of sensory events starts with the external ear and a compliant structure, the tympanum. The tympanum is set into vibrations, which ultimately lead to a neural response of the inner ear hair cells. Since the specific acoustic impedance z, which describes the resistance to movement, depends on the material’s density ρ and can be defined as the product of density and velocity of sound propagation c (air:density 1.2 kg m^−3^; velocity 343 m/s; z = 412 kg × s^−1^ × m^−2^ = 420 Pa × s × m^−1^; water:density 1000 kg m^−3^; velocity 1480 m s^−1^; z = 1.48 MPa × s × m^−1^), the enormous mismatch between air and water (inner ear) can easily be seen.

Different from specific acoustic impedance z (Pa × s × m^−1^), acoustic impedance is defined as the ratio of sound pressure p to the velocity ν of a volume of fluid (Z = p/v; acoustic volume velocity or medium flux in m^3^ s^−1^). The impedance match due to the middle ear is reached by increasing sound pressure and decreasing the volume velocity and displacement at the oval window (Mason 2016).

Without proper impedance matching, more than 99% of the acoustical energy contained in sound propagating in low-impedance air would be reflected from the inner ear, because it is filled with high impedance water (resembling sea water). The effect can readily be appreciated when considering a situation where you are under water diving and someone loudly talking to you from above the water: Very likely you don’t hear anything, because only about 0.1% of the incident energy is transmitted into the water, equivalent to a loss by 30 dB.

In mammals, it is mainly the middle ear, which helps to overcome this problem. Its three ossicles (malleus, incus, and stapes) couple airborne sound to the fluid-filled cochlear vestibule, effectively transmitting its power across the air–fluid boundary. The pressure measured at the tympanic membrane is increased nearly 20-fold, because the force acting on the relatively large diameter tympanum (human: c. 85 mm^2^; physiologically effective area c. 55 mm^2^) is focused on the much smaller oval window/stapes footplate (human: c. 3.2 mm^2^). Pressure is increased by the ratio of the two areas, which is by c. 17 times. In addition, the middle ear chain of bones causes a lever action as described above and multiplies the force by another 1.3 times. At threshold, ossicular displacements on the order of Angstrom units and forces on the order of 10^−9^ N are transduced by the ear (Rosowski 2003). Again at threshold, the vibration amplitude of air is only about 10^−11^ m at a sound pressure of 20 µPa (Fletcher 1992; Bennet-Clark 2001). These values are close to the level of ambient noise and, thus, sufficiently small from a biological point of view. Interestingly, a considerable impedance mismatch remains, only 10% of the theoretically ideal match being achieved (Moller 1974; Fletcher 1992 in Bennet-Clark 2001).

It may be appropriate to point out here that impedance matching continues down to the molecular level in the inner ear hair cells. The molecular gating spring (s.a.) is a compliant structure transmitting external forces to the transduction channels. Thus, the mechanical impedance of more rigid structures (like cytoskeleton or extracellular matrix) is matched to the more compliant gating structures of the transduction channels (Sun et al. 2019).

In many if not most cases absolute sensitivity of mechanoreceptors is not carried to an extreme in terms of the physics involved, simply because the particular animal species does not need it. In addition, optimization in biology always is a compromise based on a structure’s multifunctionality and its biologically relevant application. The sensitivity of some sensors, however, is remarkably high and it is these outstanding cases from which one hopes to learn most. Here, the “tricks” at work are expected to be most obvious. On the other hand, limiting sensitivity to keep a stimulus within the working range may be as important. The mechanical properties of the stimulus-transmitting structures contribute considerably to reach this goal. In a few cases, the underlying principles are rather well understood and point to the fundamental importance of understanding the processes of stimulus uptake and stimulus transformation preceding any neuron`s involvement.

A noteworthy simplification made here is that the middle ear also is a mechanoacoustic filter and its power transfer efficiency highly dependent on frequency. As detailed by Rosowski (2003), the external and middle ears of terrestrial vertebrates differ in size and shape, stiffness, inertance, and damping, thereby adjusting sensitivity to the frequency ranges most relevant in regard to the demands of behavior and ecology.

#### Insect ears

Obviously, there is impedance matching in *arthropod ears* as well. Again, from the acoustic properties of air and water (ratio of specific acoustic resistances 1 to 3650; sound pressure ratio 1 to 60.4), it follows that, for an efficient power transfer from air to tissue, the transformer should increase pressure 60-fold and reduce velocity 60-fold (Bennet-Clark 2001). The opposite goes for sound production and radiation, which we do not discuss here (Bennet-Clark 1995).

The compliant structure to which airborne sound has to be coupled in pressure-driven arthropod ears like those of locusts and cicadas (for velocity driven receivers s. below) is a tympanum or thin diaphragm bordering an air-filled space. The sound wave vibrates the tympanum, which, in turn, stimulates the sensory cells. A well-matched tympanum should neither be too thin not only to transmit instead of absorb sound energy, nor too thick and stiff to allow for a large enough vibration amplitude. Its mass and stiffness together will determine its efficiency, apart from its shape. Like the mammalian middle ear, the arthropod tympanum also is a mechanical filter, the low-frequency end dominated by stiffness and the high-frequency end dominated by mass. There is good evidence for frequency-dependent impedance matching in the abdominal locust ears (Stephen and Bennet-Clark 1982; Bennet-Clark 2001). Their tympana are heterogeneous regarding thickness. Four groups of sensory cells are attached to three sclerites in the posterior thin region (Müller’s organ). Membrane mechanics differs for the four groups, determining the differences in their frequency tuning (Michelsen 1971a, b; Breckow and Sippel 1985).

Adequate stimulation of the dendritic ends of the sensory cells results from the fact that Müller’s organ, which is relatively heavy and its attachment to the tympanum quite compliant, vibrates with lower amplitude and with a phase differing from that of the tympanum (Bennet-Clark 2001).

The ears of Tettigoniid grasshoppers (“bushcrickets”) are not in the abdomen, but are tympanal organs located in the proximal tibiae of the first pair of legs (e.g. Bailey 1990; Rössler et al. 2006). The main sound input does not come through the two tympana, but through an auditory trachea, which opens to the outside through the thoracic auditory spiracle (Michelsen et al. 1994). The size of this spiracle correlates with hearing sensitivity (in particular in the ultrasonic range) and also varies in different species. As found by Strauß et al. (2014), spiracle size also varies with the kind of communication in the *Poecilimon* genus: They are largest in species with bidirectional communication, where both sexes are singing and listening to each other, as opposed to unidirectional communication, where the males sing and the females approach phonotactically.

#### Arthropod medium-flow sensors

In engineering, pressure is often transformed by lever systems or hydraulic mechanisms. As stated above, such pressure and force transformation, respectively, is also realized in arthropod hair sensilla like insect filiform “hairs” and the analogous spider trichobothria. Their shafts form first-order levers amplifying force up to several hundred times on the way to the sensory cell (see above and Shimozawa and Kanou 1984; Barth et al. 1993; Shimozawa et al. 1998). Driven by the movement of the surrounding viscous fluid, these “hairs” function in air and water. They have received considerable attention regarding the mechanical” design” principles at work, building mainly on the early work by Fletcher (1978) and Shimozawa and Kanou (1984) (Barth et al. 1993; Shimozawa et al. 1998, 2003a, b; Devarakonda et al. 1996; Humphrey et al.^2001^; Humphrey and Barth 2008; Bathellier et al. 2012; Barth 2014; see also references in these papers). Both extensive experimental work and physical–mathematical modeling are now available. Knowing the dependence of the “hair’s” motion on the physical parameters that affect it helps to understand the working of impedance matching. It also allows insights in the evolutionary adaptation to the relevant physical constraints. In the following, the complexity of the task is shortly addressed.

*Number of parameters.* The parameters mainly to be addressed are hair diameter (*d*) and length (*L*), and the spring stiffness *S* (Nm/rad), representing the elastic restoring force, which resists hair deflection and brings the hair back to its equilibrium position. Furthermore, the damping constant *R* (Nms/rad) has to be considered. It is a frictional element slowing down hair motion and dissipating its energy. Finally, the density (*ρ*, kg m^−3^), viscosity (*µ*, dynamic viscosity, kg ms^−1^ or Pas or Ns /m^−2^), and velocity (V, m s^−1^) of the medium have to be taken into account (Humphrey et al. 2001, Humphrey and Barth 2008).

##### The eminent role of hair length

Although the physics and the corresponding calculations are quite complex, it seems safe to conclude that sensitivity and, thus, impedance matching is most strongly influenced by hair length *L*, followed by *R, S*, and *µ.* A 500 µm long hair in air may serve as an example (Humphrey and Barth 2008): the fractional increase in hair length needed to induce a decrease in resonance frequency is 0.5 (half) the fractional decrease required for *S*, and 0.2 the fractional increase required for *R*. This finding is of particular interest, since spider trichobothria typically occur in clusters of 2–30 hairs, which vary in length between about 0.1 mm and 1.5 mm and have preferred frequency ranges between about 40 Hz and 600 Hz. Groups of hairs differing in length by fractions of a millimeter only form sets of band-pass filters enlarging the group’s overall range of high sensitivity. It also means that the parameter needing least energy to change is indeed the one mainly used to match the most sensitive frequency range of a hair to the biologically relevant needs. A comparison of the effects of *ΔL* and *Δd* supports this conclusion. The mass change needed to change the resonance frequency is 20 times larger for *Δd* than *for ΔL*. Thus, in terms of required mass, changes in *L* produce changes in resonance frequency much more economically than changes in *d* (Humphrey et al. 2001; Barth 2002). To find out the “optimal” (perfectly matched) values of d, *L, S* and *R* for a particular stimulus and sensitivity remains an intriguing problem to solve, because cost functions and biological and ecological constraints have to be taken into account as well.

An additional aspect to be considered is the relation between hair length and boundary-layer thickness *δ*. Remarkably, the lengths of airflow-sensing hairs of widely differing arthropods like scorpions, spiders, and crickets all vary within a narrow range only, roughly between 100 µm and 2000 µm. The most effective way to adjust the mechanical frequency response of a hair is to vary its length in this range (Humphrey et al. 1993; Humphrey and Barth 2008). This is not simply due to the change in mass and hair suspension stiffness but primarily due to a match between hair length and boundary-layer thickness at the frequencies of biological relevance, which it is best matched to detect (Barth et al. 1993; Steinmann et al. 2006; Humphrey and Barth 2008).

##### Hairs in water

The outstanding relevance of hair length is again underlined when considering motion sensing hairs in water. Such hairs are common to many aquatic animals, in particular the crustaceans. In water, boundary-layer thickness is smaller than in air by a factor of 0.22, because the kinematic viscosity (dynamic viscosity/density) of water is about 20 times less than in air. In addition, the drag per unit length of the hair is 43 times larger in water due to its greater density. Finally, the virtual mass of the medium moved with the hair is much more important than in air and actually dominates the hair shaft’s inertia (Devarakonda et al. 1996; Barth 2002). A consequence of these circumstances is that one would predict that, due to the difference in density and kinematic viscosity between the two media, flow detecting hairs in water may be much shorter than in air, exhibiting the same sensitivity. Such hairs, well matched to the properties of water, seem indeed to exist. Mechanically and morphologically identical hairs are tuned to much lower frequencies in water than in air (Devarakonda et al. 1996; Barth 2002).

##### Extraordinary sensitivity

Both insect filiform hairs and spider trichobothria are strongly damped band-pass filters. Their tuning curves lack sharp peaks around their best frequency ranges (Fig. 4b). The mechanical impedance of the hairs, which are taken to be oscillating cylinders, is given by the conversion factor from air velocity to drag force (Stokes 1851). The resistance to hair deflection in their base ensures efficient energy absorption by the endings of the sensory cells (load). It seems to match well with the source (air movement) impedance, which is the frictional resistance of the air–hair contact (resulting in torque), and, thus, promotes maximum power transmission (Shimozawa et al. 1998). This then must be one of the main reasons for the extraordinary sensitivity of filiform hairs and trichobothria. At threshold, it is among the highest sensitivities found in any sensory system, indeed close to thermal noise due to Brownian motion (*k*_B_T: 4 × 10^−21^ Joules at 300°K), and clearly higher than in photoreceptors detecting a single photon (c. 3 × 10^−19^ Joules) (Thurm et al.^1983^, Barth et al.^1993^, Humphrey et al. 1993; 2003; Shimozawa et al. 2003a, 2003b, b; Humphrey and Barth 2008). In a rather broad range of frequencies, the hairs work close to the physical limit, their motion closely following that of the stimulating flow, because only a minimum of energy is dissipated by forces acting in their articulation with the exoskeleton (Humphrey and Barth 2008; Bathellier et al. 2012).

##### Energy transfer or maximal motion?

According to Shimozawa et al. (2003a, b), the internal resistance, that is the resistance of the coupling to the dendrite of the sensory cell, is matched with the frictional resistance at the air–hair contact in cricket filiform hairs. This is interpreted as an adaptation for energy sensitivity, implying optimization for energy transmission, which requires balanced damping in air and in the medium (impedance matching, r = 1). Bathellier et al. (2012) recently discussed whether spider trichobothria are optimized mechanically in regard to impedance matching (energy transfer) or maximal possible motion, which requires minimal damping in the suspension. It seems that optimization is not uniform in this regard and there may be a compromise between the two criteria (r between 0 and 1). The most interesting finding of this study is that the frequency at which angular displacement of the hair shaft is largest is suboptimal regarding the absolute physical limits (see also Barth et al. 1993). Possibly, evolution did work towards maximal sensitivity to higher frequencies rather than towards optimal responses to frequencies where angular velocity is maximal. The frequency band with minimum energy dissipation by forces acting in the hair’s articulation is beyond the frequencies at which the angular displacement is maximal (Bathellier et al. 2012). Clearly, there is food for more thought and experiment.

#### The elephant vibration sense and the Dolphin’s melon

A final example of impedance matching in mechanosensory systems is the remarkable vibration sensitivity of elephants (O’Connell-Rodwell 2007; Narins and Clark 2016). Their low-frequency vocalizations of around 20 Hz (infrasound) are strong enough to couple to the ground and travel along the surface as Rayleigh waves for miles at a velocity of about 250 m/s. Rayleigh waves attenuate by only 3 dB per doubling distance as compared to 6 dB for the spherically spreading airborne sound and are less susceptible to environmental interference. Apart from the other specializations like a large malleus in the middle ear, elephants are believed to use the dense fat contained in their foot pads (in the heel) to bridge the impedance mismatch between the ground and the elephant’s body to efficiently detect earth-borne vibrations. The foot pads might even work like “seismic lenses” to improve sensitivity (O’Connell-Rodwell et al. 2001).

One is reminded of the “acoustic fat”, a mixture mainly of triglycerides and wax esters, in the melon of dolphins and other toothed whales (Odontocetes). These melons in the forehead, with their low-velocity (low-density) core and their high-velocity (high density) shell, serve to impedance match a dolphin’s biosonar sounds from the air-filled nasal sacs to seawater, thereby avoiding their reflection back into the head. Remember the inverse problem of sound energy transmission in the hearing of terrestrial mammals and the role of the middle ear in solving the impedance mismatch between air and inner ear fluid. A difference between the two cases is the way in which an impedance match is achieved. Whereas, in our ear, the main trick is to convert low pressure (over large tympanum) to much higher pressure (over small oval window), it is a change in sound wave velocity within the melon to the much higher velocity in water (air: c.344 m s^−1^; water: 1376 m s^−1^). In toothed whales, another fat body overlying the lower jaws seems to play a similar role, being an acoustic window receiving sound in what is usually referred to as “jaw hearing”. Melons are also contributing to focus the emitted sound like an acoustic lens, probably based on the fact that changes in velocity go along with refraction, that is a change in the direction of sound propagation. (Varanasi et al.^1975^; Hughes 1999; Cranford et al. 2008; Narins and Clark 2016).

## Conclusion

- All too often the relevance of biomechanics is rather associated with dead objects. The numerous examples given in this review show how “alive” biomechanics is. It is, indeed, crucial in enabling mechanosensory organs of all subtypes to match the particular demands of their specific tasks and to cope with the animal´s specific ecological constraints and behavioral challenges. Mechanical mechanisms are profoundly involved in rendering mechanosensors as sensitive and selective as they are and have to be.
- We have also seen how powerful the role of mechanics is in stimulus uptake and transformation and how much of mechanoreceptor versatility may be due to sometimes minute differences in mechanical behavior.
- Sometimes, the mechanics at work seems to be easy to understand at first sight. However, we always have to be aware of the fact that there usually is a complex network of relations at many levels of organization. In the end, the mechanics usually is not trivial at all. We are better in taking things apart down to the molecular level than in putting them back together again and understanding all dependencies. There is hardly any mechanosensor yet, where one could be fully satisfied with the depth and completeness of the analysis of all the mechanical aspects relevant for its functioning.
- Understanding the principles of stimulus uptake and transformation by non-nervous auxiliary structures not only helps to understand the working of the sensors but also allows insights into evolutionary constraints. Much too often, these can be addressed in a very vague way only; thus, hypotheses regarding their role in adaptive radiation and the link between constraint and selection remain hard to specify.
- An important issue much neglected is the question of how efficient a sensor is in regard to its energy need. Clearly, sensory and nervous systems are very costly energy-wise (Laughlin 2001; Niven and Laughlin 2008). This implies that saving energy must have been a dominant evolutionary selection pressure. All the selectivity and sensitivity and “matching” discussed in the present review are deeply linked to the question of energy consumption, and to saving energy by focusing on the most necessary information and avoiding unnecessary over-capacity (Niven et al. 2007). Whereas big advances have been made regarding the neurons and their aggregations, in particular in regard to vision, there is hardly anything comparable for the auxiliary structures, including those of mechanoreceptors. However, clearly, representing correlation detectors, which filter out predictable input and thus extract the biologically relevant signals from a flood of irrelevant stimuli and noise, all the cases of the passive mechanical pre-processing discussed in this review greatly contribute to economical sensing.

## Footnotes

1 Viscoelasticity is a property of materials, which have both elastic and viscous properties. When stretched, they are strained in a time-dependent way. Viscoelasticity is typical of synthetic polymers, and also of ligaments and tendons. These may be damaged when loaded beyond their elastic limit and, importantly, at too high a rate of change of length. Viscoelastic materials show a hysteresis in their stress–strain curve (indicating energy loss during the loading cycle) and under constant load exhibit both stress relaxation and creep, that is increasing strain. Different well-known models, representing combinations of springs (elasticity components) and dashpots (viscosity components) in different arrangements, are applied to describe a material’s response to loading (like the Maxwell model, the Kelvin–Voigt model, and the Standard Linear Solid Model).

2 There are also examples of mismatch between sensory systems and the relevant signals and cues. As pointed out by Römer (2016), they may tell us something about the complexity of evolution, multifunctionality, conflicting selection pressures, and trade-offs. In general, one should be careful when identifying a mismatch. Thus, the tuberous electroreceptors of the weakly electric fish *Gnathonemus petersii* do not have their greatest sensitivity in the range of the electric organ discharge frequency (EOD). The EOD frequency comes to lie on the steep part of the threshold curve implying a maximized change of receptor discharge rate with the slightest change of the EOD waveform as induced by capacitive objects in the fish’s habitat. In addition to the shape of the EOD waveform the peak power spectral frequency is shifted to higher frequencies by capacitive objects. As a result, the latency of the tuberous organ’s A cells increases, while their firing rate decreases. B cells respond to waveform distortions with decreasing latency and increasing discharge frequency, when the animal is stimulated with a phase-shifted EOD. The current suggestion is that capacitive objects are sensed by a central nervous comparison of the responses of A and B cells (Von der Emde and Bleckmann 1992; von der Emde and Ruhl 2016).

3 Our attention in this review focuses on the biology of mechanosensitive sensors instead of on details of the broader physics context of the interaction between randomness and non-linearities. A wealth of literature on the definition and on misconceptions of stochastic resonance is discussed in a review article by McDonnell and Abbott (2009).

4 Arthropod medium-flow sensing hairs respond to the frictional forces transmitted by the stimulus, which, therefore, has vector properties. Whereas filiform hairs on the cerci of crickets and cockroaches (Nicklaus 1965) are highly directional due to the structure of their articulation (Gnatzy and Tautz 1980), many spider trichobothria are deflected in all horizontal directions equally or nearly equally well (Barth et al. 1993). Accordingly, just considering the sensors in the periphery, in crickets and cockroaches, the analysis of stimulus direction might rest on the activity of several hairs tuned to different directions, whereas, in spiders, a single isotropic trichobothrium might at least roughly indicate any stimulus direction. Importantly, cricket filiform hairs are supplied with a single sensory neuron only, whereas spider trichobothria are supplied by four sensory cells, each of them best responding to hair deflection in a different directional range (Görner 1965; Christian 1971; Anton and Barth 1993).
